# Restoring skeletal muscle mass as an independent determinant of liver fat deposition improvement in MAFLD

**DOI:** 10.1186/s13395-023-00333-z

**Published:** 2023-12-19

**Authors:** Ting Zhou, Junzhao Ye, Ling Luo, Wei Wang, Shiting Feng, Zhi Dong, Shuyu Zhuo, Bihui Zhong

**Affiliations:** 1 Department of Gastroenterology, The First Affiliated Hospital, Sun Yat-sen University, No. 58 Zhongshan II Road, Yuexiu District, Guangzhou, 510080 China; 2 Department of Medical Ultrasonics, Institute of Diagnostic and Interventional Ultrasound, The First Affiliated Hospital, Sun Yat-sen University, No. 58 Zhongshan II Road, Yuexiu District, Guangzhou, 510080 Guangdong China; 3 Department of Radiology, The First Affiliated Hospital, Sun Yat-sen University, No. 58 Zhongshan II Road, Yuexiu District, Guangzhou, 510080 Guangdong China; 4 Department of Nutrition, The First Affiliated Hospital, Sun Yat-sen University, No. 58 Zhongshan II Road, Yuexiu District, Guangzhou, 510080 Guangdong China

**Keywords:** Metabolic associated fatty liver disease, Relative skeletal muscle mass, Treatment response, Liver fat content, Weight loss

## Abstract

**Aims:**

Cross-sectional studies have demonstrated the association of skeletal muscle mass with metabolic-associated fatty liver disease (MAFLD), while longitudinal data are scarce. We aimed to explore the impact of changes in relative skeletal muscle mass on the MAFLD treatment response.

**Methods:**

MAFLD patients undergoing magnetic resonance imaging-based proton density fat fraction for liver fat content (LFC) assessments and bioelectrical impedance analysis before and after treatment (orlistat, meal replacement, lifestyle modifications) were enrolled. Appendicular muscle mass (ASM) was adjusted by weight (ASM/W).

**Results:**

Overall, 256 participants were recruited and divided into two groups: with an ASM/W increase (*n*=166) and without an ASM/W increase (*n*=90). There was a great reduction in LFC in the group with an ASM/W increase (16.9% versus 8.2%, *P* < 0.001). However, the change in LFC in the group without an ASM/W increase showed no significant difference (12.5% versus 15.0%, *P* > 0.05). △ASM/W _Follow-up-Baseline_ [odds ratio (OR)=1.48, 95% confidence interval (CI) 1.05-2.07, *P* = 0.024] and △total fat mass (OR=1.45, 95% CI 1.12-1.87, *P* = 0.004) were independent predictors for steatosis improvement (relative reduction of LFC ≥ 30%). The subgroup analysis showed that, despite without weight loss, decrease in HOMA-IR (OR=6.21, 95% CI 1.28-30.13, *P=*0.023), △total fat mass _Baseline -Follow-up_ (OR=3.48, 95% CI 1.95-6.21, *P* <0.001 and △ASM/W _Follow-up-Baseline_ (OR=2.13, 95% CI 1.12-4.05, *P*=0.022) independently predicted steatosis improvement.

**Conclusions:**

ASM/W increase and loss of total fat mass benefit the resolution of liver steatosis, independent of weight loss for MAFLD.

**Supplementary information:**

The online version contains supplementary material available at 10.1186/s13395-023-00333-z.

## Introduction

Metabolic-associated fatty liver disease (MAFLD), a new nomenclature renamed nonalcoholic fatty liver disease (NAFLD) [1], has become a predominant cause of chronic liver disease globally. Paralleling the obesity epidemic, MAFLD affects an estimated 38.77% of the general population worldwide according to a recent meta-analysis [2]. MAFLD encompasses pathologic traits from simple steatosis to steatohepatitis and fibrosis, which continue to progress to cirrhosis or hepatocellular carcinoma, a recent meta-analysis reported that the annual incidence of hepatocellular carcinoma was 1·8 cases per 1000 person-years in patients with MAFLD [3]. Moreover, numerous clinical studies have demonstrated that MAFLD is closely associated with higher risks of extrahepatic diseases, including type 2 diabetes mellitus (T2DM), cardiovascular disease and chronic kidney diseases [4]. However, there is no approved pharmacotherapy internationally for treating MAFLD to date [1].

Skeletal muscle has been recognized as an important endocrine organ responsible for glucose utilization facilitated by insulin, and the loss of skeletal muscle mass may reduce insulin-mediated glucose disposal [5]. Sarcopenia is characterized by the severe loss of skeletal muscle mass accompanied by progressively reduced muscle strength and physical performance [6]. Mounting studies have demonstrated that lower skeletal muscle mass or sarcopenia is correlated with the prevalence of MAFLD [7–11], more severe steatosis [10, 12–15] and significant fibrosis [12–17] confirmed by biopsy or noninvasive examination, such as transient elastography. Nevertheless, longitudinal studies investigating the relationship between low muscle mass and the severity of steatosis as well as fibrosis in MAFLD are limited. Moreover, the effect of skeletal muscle mass on the MAFLD treatment response is not fully understood. Loss of skeletal muscle mass has been reported to cause metabolic impairments and further aggravate MAFLD [18]; however, evidence regarding the association between changes in skeletal muscle mass and the progression or improvement of MAFLD is scarce.

Magnetic resonance imaging-based proton density fat fraction (MRI-PDFF) is a quantitative and accurate method that is used to assess liver fat content (LFC) [19]. MRI-PDFF has been proven to have excellent diagnostic value for LFC and histologic steatosis in MAFLD patients [20] and is more sensitive to longitudinal changes in steatosis than biopsy [21]. Therefore, MRI-PDFF is regarded as a precise technique for assessing the severity of steatosis and can be utilized in clinical studies to evaluate the relative change in LFC [22, 23].

In this study, we aimed to determine (1) the association of relative skeletal muscle mass or its changes measured by bioelectrical impedance analysis (BIA) with the changes in severity of steatosis and fibrosis in MAFLD patients and (2) the influence of changes in skeletal muscle mass on liver function and insulin resistance based on longitudinal research.

## Materials and methods

### Study participants

This was a prospective single-center observational cohort study conducted in the First Affiliated Hospital, Sun Yat-sen University, from January 2017 to August 2022. The study design was approved by the institutional ethics committee for clinical research of the First Affiliated Hospital, Sun Yat-sen University (Approval number: [2014] 112). All procedures performed in this study involving human participants were in accordance with the ethical standards of the institutional research committee and with the Helsinki Declaration of 1975. All patients signed written informed consent forms.

The subjects were consecutively enrolled according to the following inclusion criteria: (a) age ≥ 18 years; (b) diagnosed with MAFLD based on the international expert consensus in 2020 [24]; and (c) underwent at least two examinations with MRI-PDFF and bioelectrical impedance analysis (BIA). All participants were required to complete a face-to-face interview questionnaire regarding demographic information, smoking, alcohol consumption, medical history and drug use. The following exclusion criteria were applied: (a) hepatocellular carcinoma based on imaging evidence; (b) decompensated cirrhosis; (c) hepatitis B (hepatitis B surface antigen positive for over six months) and hepatitis C (positive for hepatitis C antibody) infection; (d) excessive alcohol consumption (> 20 g/week for males or > 10 g/week for females); (e) auto-immune hepatitis; (f) coronary heart disease, heart failure, chronic kidney disease and malignancies; (g) pregnancy and breastfeeding; and (h) use of drugs that induce steatosis such as steroids, amiodarone or tamoxifen.

### Clinical and laboratory indices

Anthropometric indices, including weight, height, waist circumference and blood pressure, were measured by two well-trained physicians. Body mass index (BMI) was calculated as weight/height^2^ (kg/m^2^). After a 12-hour overnight fast, a venous blood sample was drawn from all patients and subsequently tested by the laboratory. Biochemical parameters, including alanine aminotransferase (ALT), aspartate aminotransferase (AST), γ-glutamyl transpeptidase GGT), alkaline phosphatase (ALP), total lipid profiles, fasting serum glucose (FSG), fasting insulin (FIN) and uric acid (UA), were assayed by the Abbott c8000 Automatic Biochemistry Analyzer (Abbott, Abbott Park, IL, USA). The normal upper limit for ALT was set to 30 U/L for males and 19 U/L for females [25]. Obesity was defined as BMI ≥ 25kg/m^2^ [26]. Homeostasis model assessment of insulin resistance (HOMA-IR) was calculated using the following equation: HOMA-IR = FSG (mmol/L) *FIN (μU/mL)/22.5 [24]. Insulin resistance was defined as HOMA-IR ≥2.5 [27].

### Radiology examination

MRI-PDFF of the upper abdomen was conducted with a 3.0-Tesla MRI scanner (SIEMENS 3.0T MAGNETOM Verio, Siemens, Munchen, Germany) in all participants for LFC quantification at baseline and follow-up visits. The scanning protocol and imaging parameters were in accordance with those of our previous published study [28]: TE1 2.5 ms; TE2 3.7 ms; repetition time 5.47 ms; 5° flip angle; ±504.0 kHz per pixel receiver bandwidth; and slice thickness, 3.0 mm. The LFC was evaluated with an irregularly shaped region of interest covering the entire liver in 21 sequential slices by two trained radiologists who were blinded to the aim of the study.

The liver stiffness measurement (LSM) was performed by two-dimensional shear wave elastography (2D-SWE, Aix-en-Provence, France) at the first clinic visit and follow-up. The physicians who conducted the 2D-SWE had over 5 years of experience with ultrasound measurement.

### Body composition measurements and skeletal muscle mass measurements

The BIA was utilized to assess the body composition of all participants with a segmental multifrequency BIA device (TANITA, MC-980MA, Japan) according to the manufacturer’s instructions. The BIA technique showed good correlation with dual-energy X-ray absorptiometry, which was validated for the assessment of body composition [29, 30]. The patients were instructed to stand on the electrodes under the toes and heels, and hold a handle in each hand, after fasted overnight (at least 8 hours). All participants spread apart their limbs to ensure that their arms didn’t touch the trunk and the thighs were not in contact, remaining motionless for 40 seconds during the measurement. The impedance for each segments including four limbs and the trunk were measured and the device calculated skeletal muscle mass by regression equations developed by Yamada et al [31]. The appendicular skeletal muscle mass (ASM) was calculated by the sum of the lean muscle mass of the upper and lower limbs. The skeletal muscle mass index (SMI) was calculated by dividing the ASM by body weight (kg), expressed as a percentage (ASM/body weight × 100%).

### Treatment and follow-up

Patients received orlistat intervention, meal replacement or routine treatment at baseline and maintained it until the last follow-up. Subjects who chose the orlistat intervention received orlistat (120 mg, 3 times/day for 24 weeks) without additional treatment. Orlistat administration was confirmed by prescription and recorded during clinic visits, as described in our previous study [23]. Those who chose meal replacement were given a nutrient composition of 40% to 50% of calories from carbohydrates, 20% to 35% from protein, and 25% to 30% from fat, which was provided by Jintong Special Medical Food Co., Ltd. (Guangzhou, China), without additional treatment. Patients who refused orlistat or meal replacement and received lifestyle modifications were instructed to restrict carbohydrate and fat intake in daily life as well as to exercise 3 times a week, 30 minutes each term, according to the World Health Organization Global Strategy on Diet, Physical Activity and Health [32]. Every patient was provided with a portable manual with personalized dietary and exercise suggestions based on sex, age, BMI and medical history. In regard to patients with therapy indications for FSG and lipid profiles, drug treatments were added as guideline recommendations [33, 34]. Patients were instructed to complete two visits over the 6-month enrollment period. Clinical and laboratory parameters were collected and BIA, MRI-PDFF and 2D-SWE were performed at baseline and follow-up. The baseline LFC was compared with that at the follow-up visit. The change in LFC was calculated by subtracting follow-up LFC from LFC at baseline visit (△LFC _Baseline-Follow-up_). The improvement of steatosis was defined as a relative reduction of liver fat fraction with MRI-PDFF ≥ 30% (△LFC= LFC _Baseline-Follow-up_/ LFC _Baseline_ ≥ 30%) [35]. The change in SMI was calculated as △ASM/W _Follow-up-Baseline_.

### Statistical analysis

The quantitative variables were expressed as the mean ± standard deviation (SD) when they followed a normal distribution; otherwise, they were presented as the median (interquartile range). The baseline characteristics were compared with the independent Student’s *t* test or nonparametric test for continuous variables and the chi-square test for categorical variables. Pairwise t tests were applied to compare parameters at baseline and those at follow-up. Univariate and multivariate logistic regression analyses were utilized to determine independent associations between either baseline ASM/W or change in ASM/W and the improvement of steatosis. The factors which were significant in univariate logistic regression analysis were included in the multivariate logistic regression analysis. We also conducted a subgroup analysis defined by the change in weight and baseline BMI (BMI ≥ 25kg/m^2^, <25 kg/m^2^). Correlations of changes in ASM/W with changes in LFC and HOMA-IR were performed with Spearman’s correlation analysis. In addition, receiver operating characteristic (ROC) curve analysis was used to test the predictive value of the change in ASM/W for the improvement of ALT, LFC and HOMA-IR. The Youden index was applied to determine the cut-off values that maximized sensitivity and specificity. Two-sided *P* values < 0.05 were considered as statistically significant. Statistical analyses were performed with IBM SPSS statistical software (version 25.0, Chicago, IL, USA) and GraphPad Prism 8 (Inc, USA).

## Results

### Baseline characteristics of the study population

**Fig. 1:**
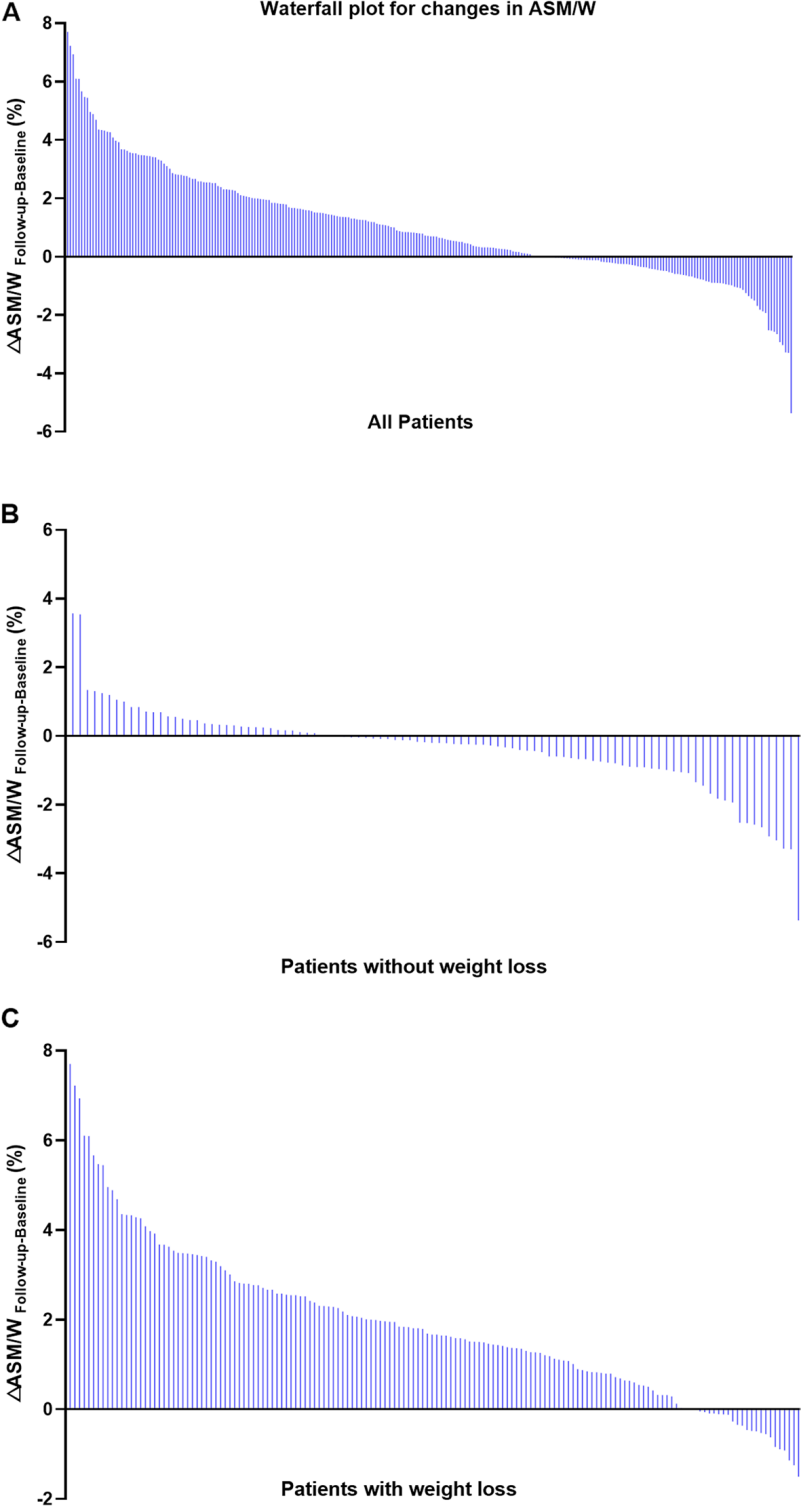
Waterfall plot of change in ASM/W from baseline in all (**A**) patients and the subgroups without and with weight loss (**B, C**). △ASM/W =ASM/W _Follow-up-Baseline_

A total of 301 patients were enrolled initially, and they were excluded for reasons such as decompensated cirrhosis (*n*=3), hepatitis B surface antigen positivity (*n*=30), and excess alcohol intake (*n*=12). Therefore, 256 MAFLD patients were ultimately recruited, consisting of 146 (57.0%) subjects who showed improvement at follow-up and 110 (43.0%) subjects who did not. In this study, 16, 10 and 230 patients chose orlistat, meal replacement and lifestyle modifications. The subgroup analysis based on the treatment suggested that the patients in lifestyle treatment gained the significant ASM/W (%) increase (32.2±4.1 vs 33.4±4.3, *P*<0.001) and the improvement of liver steatosis [14.9 (9.0, 22.7) vs 9.0 (5.9, 15.0), *P*<0.001; Supplementary table 1]. All 256 participants were divided into two groups based on the change in ASM/W, with an increase in ASM/W (△ASM/W Follow-up-Baseline >0) and without an increase in ASM/W (△ASM/W _Follow-up-Baseline_ ≤0). Figure 1 shows the change in ASM/W from baseline in all patients and in the subgroups without and with weight loss. The maximum and minimum △ASM/W _Follow-up-Baseline_ were 7.70%, 3.57%, 7.70%, and -5.36%, -5.36% and -1.50%, respectively. The baseline characteristics of the subjects are presented in Table 1. There were no significant differences in demographics, anthropometry, metabolic profiles, liver stiffness, body composition, intervention or drug use between the two groups. Interestingly, patients with an increase in ASM/W had worse liver function than patients without an increase in ASM/W, such as higher ALT, AST and GGT (all *P* < 0.05), and more severe hepatic steatosis with higher LFC (*P* < 0.001) at baseline.

**Table 1:** Comparison of the baseline characteristics between patients with and without increase in ASM/W*

Characteristics	MAFLD patients			*P*
	All (*n*=256)	With ASM/W increase (*n*=168)	Without ASM/W increase (*n*=88)	
**Demographic**				
**Age(years)**	41.1±14.4	40.9±15.1	41.4±12.9	0.79
**Male, n (%)**	182 (71.1)	121 (72.0)	61 (69.3)	0.65
**Smoke**	39 (15.2)	25 (14.8)	14 (15.9)	0.83
**Alcohol consumption**	27 (10.5)	17 (10.1)	10 (11.4)	0.76
**Anthropometric**				
**Weight (kg)**	74.1±13.7	74.4±13.3	73.5±14.5	0.61
**BMI (kg/m**^**2**^**)**	26.7±3.9	26.8±3.6	26.5±4.3	0.58
**WC (cm)**	91.9±9.5	91.6±9.2	92.5±10.2	0.48
**Diastolic blood pressure (mmHg)**	129±17	129±17	129±16	0.93
**Systolic blood pressure (mmHg)**	85±12	85±12	85±13	0.70
**Liver biochemistry**				
**ALT (U/L)**	45(29, 75)	53(31, 83)	37(21, 55)	<0.001
**AST (U/L)**	34(23, 46)	37(26, 51)	28(21, 38)	<0.001
**GGT (U/L)**	43(28, 72)	48(32, 78)	33(24, 50)	<0.001
**ALP (U/L)**	77(66, 88)	78(67, 89)	74(64, 85)	0.08
**Metabolic characteristics**				
**TC (mmol/L)**	5.0±1.0	5.0±1.0	5.0±0.8	0.71
**TG (mmol/L)**	1.5(1.2, 2.2)	1.6(1.2, 2.4)	1.4(1.2, 2.1)	0.22
**HDL-C (mmol/L)**	1.1±0.2	1.1±0.3	1.1±0.2	0.81
**LDL-C (mmol/L)**	3.1±0.7	3.1±0.8	3.1±0.7	0.78
**FSG (mmol/L)**	5.2±1.3	5.2±1.4	5.2±1.1	0.74
**HOMA-IR**	2.5(1.6, 3.8)	2.7(1.7, 4.1)	2.2(1.5, 3.4)	0.10
**Uric acid (μmol/L)**	430±110	426±109	439±110	0.38
**Metabolic syndrome**	87 (33.9)	54 (32.1)	33 (37.5)	0.39
**Liver stiffness (kPa)**	5.9(5.3, 6.8)	5.9(5.3, 7.1)	5.9(5.4, 6.6)	0.88
**LFC (%)**	14.9(9.6, 22.4)	17.1(11.2, 24.8)	11.5(7.7, 18.9)	<0.001
**Body composition**				
**ASM (kg)**	23.8±5.6	23.9±5.6	23.8±5.6	0.85
**ASM/W (%)**	32.0±4.1	31.8±4.0	32.3±4.3	0.37
**Body muscle mass (kg)**	25.9±3.9	26.0±3.9	25.7±4.1	0.68
**Total fat mass (kg)**	21.0±8.8	20.7±7.7	21.7±10.5	0.40
**Interventions**				0.64
**Orlistat**	16 (6.3)	8 (4.8)	8 (9.1)	
**Meal replacement**	10 (3.9)	4 (2.4)	6 (6.8)	
**Lifestyle**	230 (89.8)	154 (91.7)	76 (86.4)	
**Drugs**				
**Statin, n (%)**	66 (25.8)	41 (24.4)	25 (28.4)	0.23
**Antidiabetic, n (%)**	24 (9.3)	13 (7.7)	11 (12.5)	0.21

Continuous variables are reported as mean ± standard deviation (SD) or median (interquartile range). *MAFLD* metabolic associated fatty liver disease, *ASM* appendicular skeletal mass, *ASM/W*, *ASM*/weight, *BMI* body mass index, *WC* Waist circumference, *ALT* Alanine aminotransferase, *AST* Aspartate aminotransferase, *GGT* γ-glutamyl transpeptidase, *ALP* alkaline phosphatase, *TC* total cholesterol, *TG* triglyceride, *HDL-C* high-density lipoprotein cholesterol, *LDL-C* low-density lipoprotein cholesterol, *HOMA-IR* homeostatic model assessment of insulin resistance, *FSG* fasting serum glucose, *LFC* liver fat content. ^*^With and without ASM/W increase was defined as △ASM/W _Follow-up-Baseline_ >0 and ≤0, respectively

### Changes in anthropometric, biochemical, and hepatic status with or without ASM/W increase

**Fig. 2:**
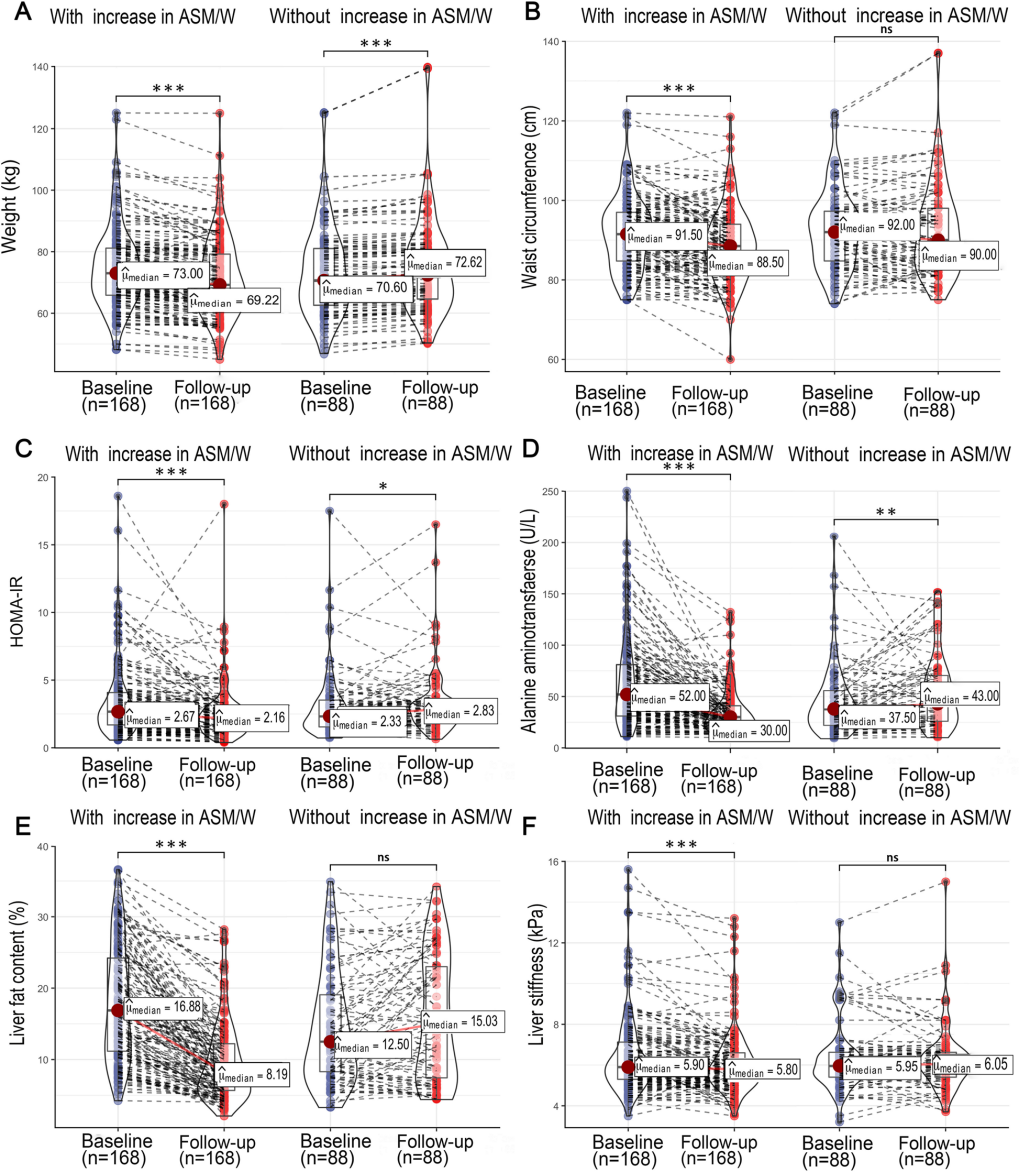
Changes in weight (**A**), waist circumference (**B**), homeostatic model assessment of insulin resistance (**C**), alanine aminotransferase (**D**), liver fat content (**E**), and liver stiffness (**F**) in groups with or without ASM/W increase. ASM, appendicular skeletal mass; ASM/W, ASM/weight; HOMA-IR, homeostatic model assessment of insulin resistance. The ASM/W increase was determined as △ASM/W>0. △ASM/W =ASM/W _Follow-up-Baseline_. The numbers on the top of the boxes represent medians. *P<0.05; **P<0.01; ***P<0.001; ns, not significant

By design, at follow-up, a significant decrease in weight and waist circumference was observed in the group with an increase in ASM/W (both *P* < 0.001), while a significant increase in weight (*P* < 0.001) and no significant changes in waist circumference (*P* > 0.05) were observed in the group without an increase in ASM/W (Fig. 2A and B). In the group with ASM/W elevation, insulin resistance and liver function improved at follow-up compared with those at baseline with a decrease in HOMA-IR and ALT (both *P* < 0.001) although the liver function at baseline was worse in this group; however, no similar improvements were found in the group without ASM/W elevation (Fig. 2C and D). In spite of a worse steatosis at baseline, the LFC and liver stiffness at follow-up were reduced significantly compared with those at baseline only in the group with an increase in ASM/W (both *P* < 0.001); however, there were no significant differences in the group without an increase in ASM/W, despite a lower LFC at baseline (Fig. 2E, F).

### Subgroup analysis divided by weight, ASM and ASM/W change

To determine the effect of change in weight, ASM/W and ASM on the improvement of steatosis, all patients were performed subgroup analysis according to with weight loss (WL), an increase in ASM/W (ASM/WI) and ASM (ASMI) or not. With WL (WL^+^) was defined as △Weight _Baseline-Follow-up_ >0 and without WL (WL^-^, -:referred to without) as △Weight _Baseline-Follow-up_ ≤0. With ASM/WI (ASM/WI^+^, +:referred to with) and without ASM/WI (ASM/WI^-^) were defined as △ASM/W _Follow-up-Baseline_ >0 and ≤0. With ASMI (ASMI+) and without ASMI (ASMI-) were defined as △ASM _Follow-up-Baseline_ >0 and ≤0. There were three classifications, each consisting of 4 subgroups: ① without WL and without ASM/WI (WL^-^ASM/WI^-^), without WL and with ASM/WI (WL^-^ASM/WI^+^), with WL and without ASM/WI (WL^+^ ASM/WI^-^); with WL and ASM/WI (WL^+^ ASM/WI^+^); ② without ASMI and ASM/WI (ASMI^-^ASM/WI^-^), without ASMI and with ASM/WI (ASMI^-^ASM/WI^+^), with ASMI and without ASM/WI (ASMI^+^ ASM/WI^-^); with ASMI and ASM/WI (ASMI^+^ ASM/WI^+^); ③ without WL and without ASMI (WL^-^ASMI^-^), without WL and with ASMI (WL^-^ASMI^+^), with WL and without ASMI (WL^+^ ASMI^-^); with WL and ASMI (WL^+^ ASMI^+^). The comparison of baseline and follow-up characteristics in four groups were shown in Supplementary table 2, 3 and 4.

**Fig. 3:**
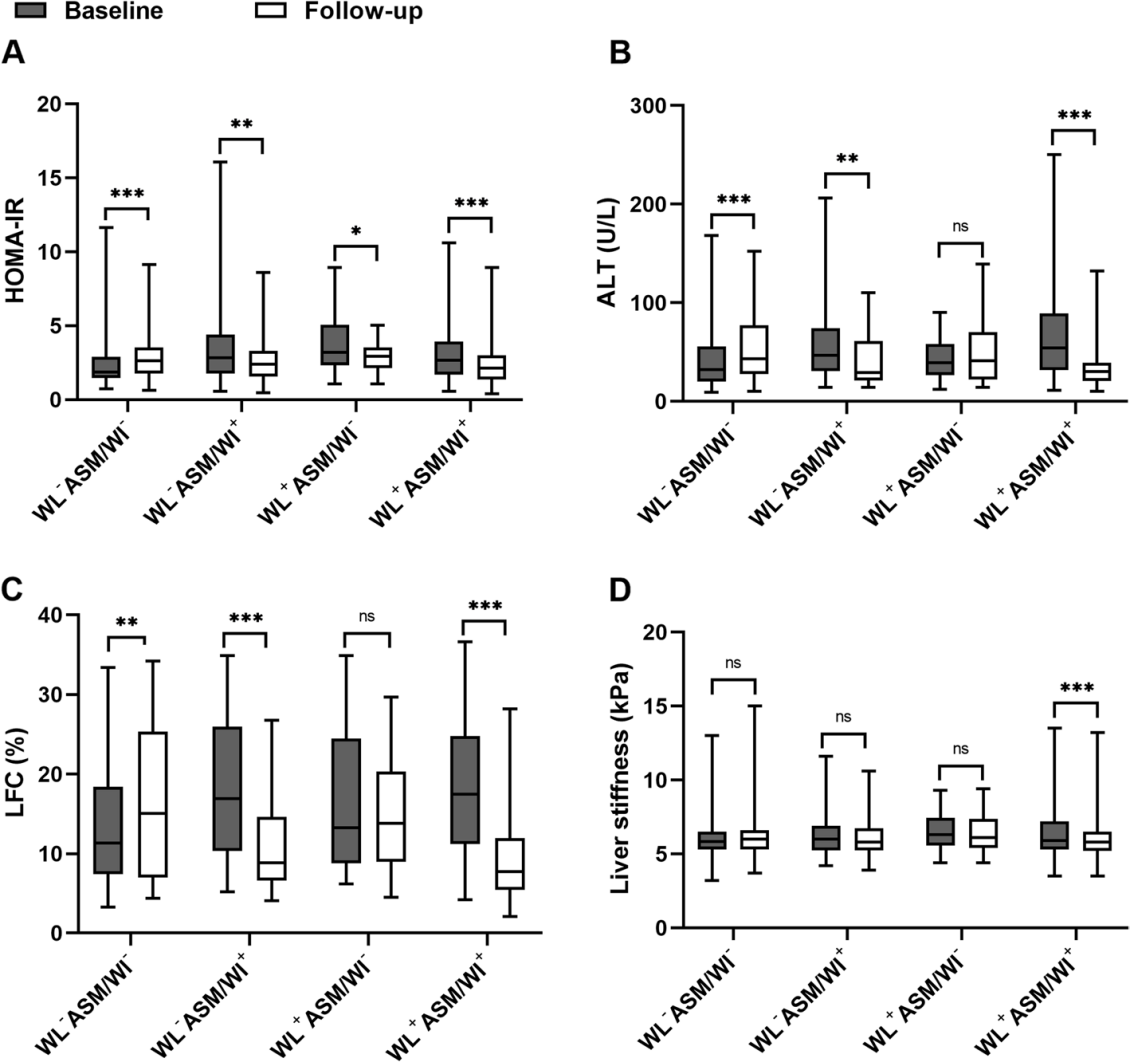
Changes in HOMA-IR (**A**), ALT (**B**), LFC (**C**), liver stiffness (**D**) in the groups classified by changes in weight and ASM/W. ASM, appendicular skeletal mass; ASM/W, ASM/weight; HOMA-IR, homeostatic model assessment of insulin resistance. Weight loss was defined as △weight>0. △weight=weight _Baseline-Follow-up_. The ASM/W increase was determined to be △ASM/W>0. △ASM/W =ASM/W _Follow-up-Baseline_. WL-, without weight loss; WL+, with weight loss; ASM/WI-, without ASM/W increase; ASM/WI +, with ASM/W increase. *P<0.05; **P<0.01; ***P<0.001; ns, not significant

A significant reduction in HOMA-IR (2.6 verse 2.2, *P*<0.001), ALT (53U/L verse 29U/L, *P*<0.001), LFC (16.5% verse 7.9%, *P*<0.001) and liver stiffness (5.9 kPa verse 5.8kPa, *P*<0.001) at follow-up compared with those at baseline in the WL^+^ ASM/WI^+^ group (Fig. 3A, B, C, D). Nevertheless, only LFC decreased significantly in the WL^-^ASM/WI^+^ group (17.3% verse 8.8%, *P*=0.001; Fig. 3C). There is also a significant improvement in HOMA-IR (2.5 verse 1.9, 2.7 verse 2.3, both *P*<0.001), ALT (54U/L verse 30U/L, 51U/L verse 30U/L, both *P*<0.001) and LFC (15.3% verse 7.4%, 17.4% verse 8.3%, both *P*<0.001) at follow-up compared with those at baseline in the ASMI^-^ ASM/WI^+^ and ASMI^+^ ASM/WI^+^ groups (Supplementary figure 1A, B, C). Similarly, significant improvement in HOMA-IR (2.7 verse 2.1, 2.7 verse 2.3, both *P*<0.001), ALT (49U/L verse 31U/L, 53U/L verse 30U/L, both *P*<0.001), LFC (16.5% verse 7.9%, *P*<0.001) were observed in WL^+^ASMI^-^ and WL^+^ASMI^+^ groups (Supplementary figure 2A, B, C). In WL^-^ASMI^+^ group, LFC reduced significantly (13.3% verse 8.8%, *P*<0.001) even though without improvement in HOMA-IR, ALT and liver stiffness (Supplementary figure 2A, B, C, D).

### Predictors of steatosis improvement

**Fig. 4:**
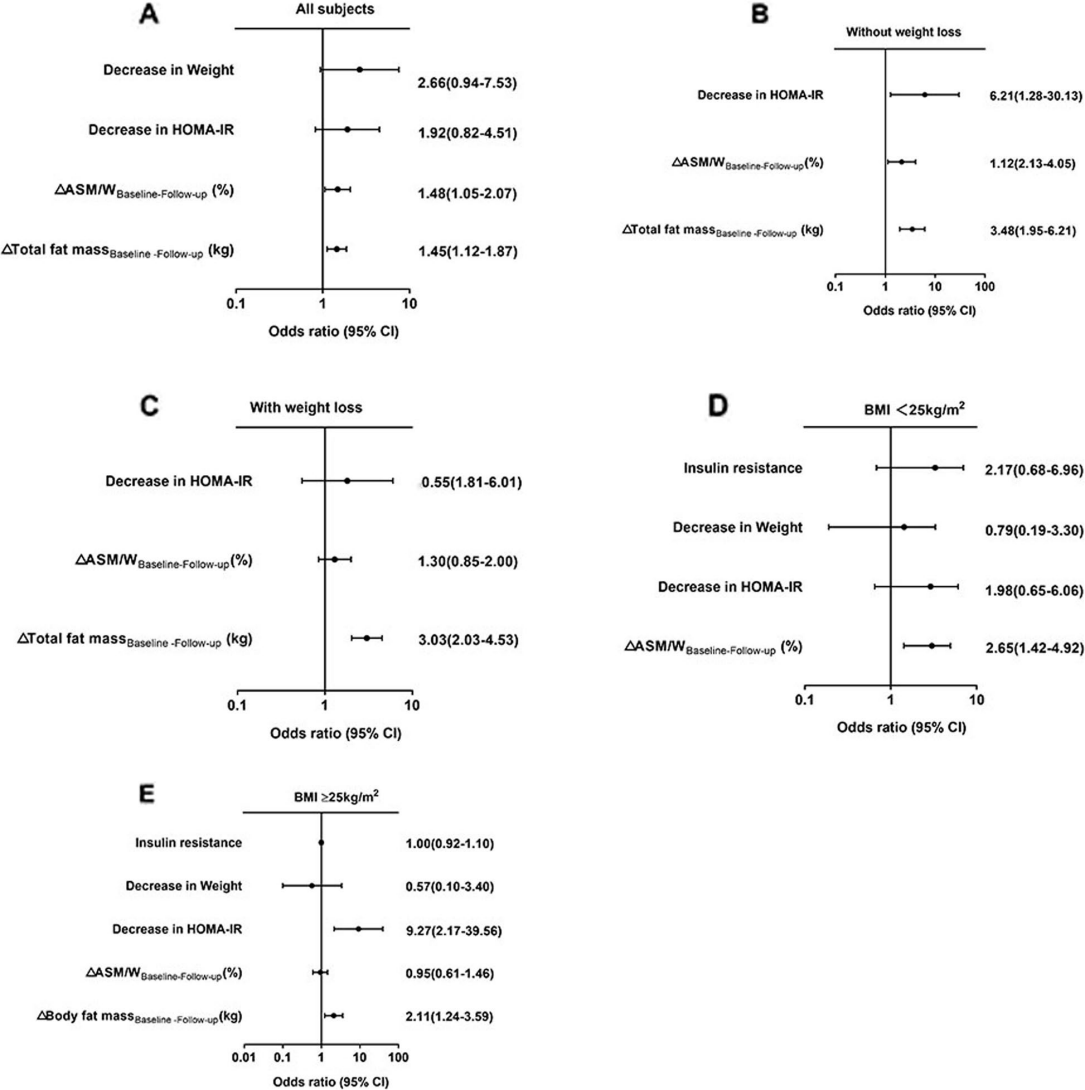
The predictors of improvement of liver fat content in univariate and multivariate logistic regression analysis in all subjects (**A**), patients without and with weight loss (**B, C**), patients with a BMI <25kg/m^2^ and BMI ≥25kg/m^2^ (**D, E**). The improvement of liver steatosis was defined as the liver fat content (LFC) ≥30% relative decline compared with baseline in MRI-PDFF (LFC _Baseline-Follow-up_/ LFC _Baseline_≥30%). ASM, appendicular skeletal mass; ASM/W, ASM/weight; HOMA-IR, homeostatic model assessment of insulin resistance. Decrease in Weight was defined as △Weight _Baseline-Follow-up_ >0; Decrease in HOMA-IR was defined as △HOMA-IR _Baseline-Follow-up_ >0

We analyzed the factors in the binary logistic regression model of liver steatosis improvement (relative reduction of LFC ≥ 30%). For all patients, decrease in weight, decrease in HOMA-IR, △ASM/W _Follow-up-Baseline_ and △Total fat mass _Baseline -Follow-up_ were identified as predictors of hepatic steatosis improvement in univariate analysis (supplementary table 5). After multivariate analysis, △ASM/W _Follow-up-Baseline_ [odds ratio (OR)=1.48, 95% confidence interval (CI) 1.05-2.07, *P* = 0.024] and △Total fat mass _Baseline -Follow-up_ (OR=1.45, 95% CI 1.12-1.87, *P* = 0.004) were found to be predictors of the improvement in liver steatosis (Fig. 4A). However, ASM/W and total fat mass at baseline and the different treatments were not significantly associated with liver steatosis remission.

Logistic analysis was conducted in the subgroups divided by weight loss, which was designed to explore the impact of weight loss on the hepatitis steatosis and ASM/W, the univariate analysis was shown in Supplementary table 5. In patients with weight loss, 113 (72.4%) achieved hepatic steatosis remission at follow-up, multivariate logistic regression analysis showed that △total fat mass _Baseline -Follow-up_ (OR=3.03, 95% CI 2.03-4.53, *P*<0.001) for predicting improvement of fat infiltration of liver (Fig. 4C). However, in patients without weight loss, 33 (33.0%) patients in this group showed improvement of hepatic steatosis, in addition to decrease in HOMA-IR (OR=6.21, 95% CI 1.28-30.13, *P=*0.023) and △total fat mass _Baseline -Follow-up_ (OR=3.48, 95% CI 1.95-6.21, *P* <0.001), △ASM/W _Follow-up-Baseline_ (OR=2.13, 95% CI 1.12-4.05, *P*=0.022) was observed as the independent predictor of steatosis improvement (Fig. 4B).

Subgroup analysis determined by obesity (BMI ≥ 25 kg/m^2^ and BMI < 25 kg/m^2^) was also performed to explore the factors associated with steatosis improvement. The baseline characteristics of the two subgroups are shown in Supplementary table 6 and the univariate analysis was shown in supplementary table 7. At follow-up, 50 (55.6 %) and 96 (57.8 %) subjects showed improvement in the group without and with obesity. In patients without obesity, multivariate logistic regression analysis after adjustment cofounders showed that △ASM/W _Follow-up-Baseline_ (OR=2.65, 95% CI 1.42-4.92, *P*=0.020) was an independent predictor of steatosis improvement (Fig. 4D). Among patients with obesity, decrease in HOMA-IR (OR=9.27, 95% CI 2.17-39.56, *P=*0.003) and △body fat mass _Baseline -Follow-up_ (OR=2.11, 95% CI 1.24-3.59, *P* = 0.006) remained significant predictors for the remission of steatosis (Fig. 4E). Subgroup analysis defined by BMI ≥ 30 kg/m^2^ and BMI < 30 kg/m^2^) was also conducted. Multivariate logistic regression analysis showed that △ASM/W _Follow-up-Baseline_ (OR=3.06, 95% CI 2.14-5.64, *P*<0.001) independently predicted steatosis improvement in subjects with BMI < 30 kg/m^2^, while could not predict in patients BMI ≥ 30 kg/m^2^ (Supplementary table 8).

### Correlation between the change in ASM/W and the change in LFC and HOMA-IR

**Fig. 5:**
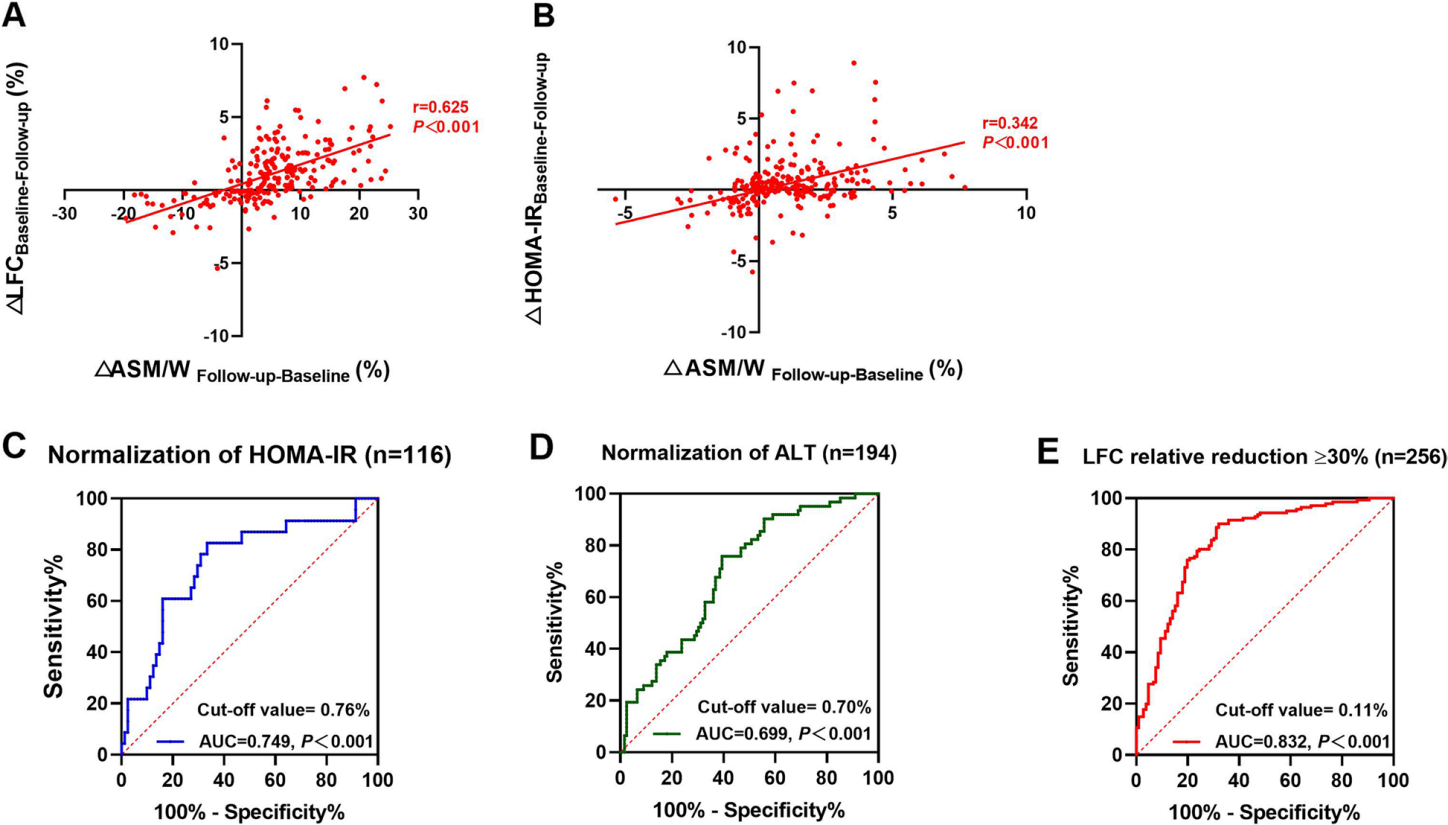
Correlation of △ASM/W with △LFC (**A**) and △HOMA-IR (**B**) in all patients. HOMA-IR, homeostatic model assessment of insulin resistance; ALT, alanine aminotransferase, LFC, liver fat content; ASM, appendicular skeletal mass; ASM/W, ASM/weight. Receiver operating characteristic curve of △ASM/W for predicting normalization of HOMA-IR in subjects with insulin resistance at baseline (*n*=116, **C**), normalization of ALT in subjects with elevation of ALT at baseline (*n*=194, **D**), △LFC reduction ≥30% in the entire cohort (*n*=256, **E**). △ASM/W=ASM/W _Follow-up-Baseline_; △HOMA-IR=HOMA-IR _Baseline-Follow-up_; △LFC= LFC _Baseline-Follow-up_. Insulin resistance was defined as a homeostasis model assessment of insulin resistance ≥2.5, and normalization of HOMA-IR was defined as a homeostasis model assessment of insulin resistance<2.5 at follow-up in this cohort. Elevation of ALT was defined as alanine aminotransferase ≥30 U/L in males and ≥19 U/L in females, and the normalization of ALT was determined when alanine aminotransferase <30 U/L in males and <19 U/L in females at follow-up in this cohort. LFC relative reduction was calculated as relative △LFC _Baseline-Follow-up_/ LFC _Baseline_

The associations of △ASM/W _Follow-up-Baseline_ with △LFC _Baseline-Follow-up_ and △HOMA-IR _Baseline-Follow-up_ were analyzed using Spearman’s correlation analysis. The △ASM/W _Follow-up-Baseline_ was positively related to △LFC _Baseline-Follow-up_ (r=0.576, *P*<0.001) and △HOMA-IR _Baseline-Follow-up_ (r=0.330, *P*<0.001, Fig. 5A, B).

### Predictive values of △ASM/W _Follow-up-Baseline_ for MAFLD treatment outcomes

We constructed an ROC curve to evaluate the predictive power of △ASM/W _Follow-up-Baseline_ for normalization of HOMA-IR in subjects with insulin resistance at baseline (*n*=116), normalization of ALT in patients with elevated ALT at baseline (*n*=194) and △LFC reduction ≥30% in the entire cohort (*n*=256). The areas under the curves (AUCs) for normalization of HOMA-IR (Fig. 5C), normalization of ALT (Fig. 5D) and steatosis improvement (Fig. 5E) were 0.749 (95% CI 0.631-0.866, *P*<0.001), 0.699 (95% CI 0.623-0.786, *P*<0.001) and 0.832 (95% CI 0.779-0.885, *P*<0.001), respectively. The cut-off values of △ASM/W _Follow-up-Baseline_ were 0.76%, 0.70% and 0.11%, with sensitivities of 75.8%, 82.6 and 86.9% and specificities of 39.3%, 33.3% and 30.5%, respectively.

## Discussion

In this prospective longitudinal study, we first explored the relationship between the change in ASM/W and the improvement of MAFLD evaluated by MRI-PDFF in Asian adults with MAFLD. We observed that the increase in ASM/W and decrease in total fat mass were independently associated with the remission of hepatic steatosis, accompanied by significant improvements in insulin sensitivity and liver function in the group with an increase in ASM/W. These associations persisted after further adjustment for change in weight.

Several previous studies have reported that a lower SMI was associated with more severe liver steatosis evaluated by ultrasonography or liver histology [10, 12, 13, 36] but were limited to cross-sectional settings. Data focused on the relationship of the baseline SMI and change in SMI with MAFLD are scarce. A 10-year retrospective cohort study involving 4398 initially MAFLD-free subjects suggested that a progressive increase in fat mass and a loss of ASM/W were associated with incident MAFLD diagnosed by ultrasonography [37]. A 7-year longitudinal study including 12624 subjects reported that the baseline SMI and an increase in SMI were positively associated with the improvement of MAFLD determined by the hepatic steatosis index (HSI) if present at baseline [27]. In line with this, an association between an increase in ASM/W and the resolution of hepatic steatosis determined by a relative reduction in LFC obtained by MRI-PDFF was observed in this study. In addition, the logistic regression analysis suggested that this association was independent of weight change. ROC analysis showed that the cut-off value of △ASM/W _Follow-up-Baseline_ for predicting steatosis improvement was 0.14%. Moreover, we also found that a decrease in total fat mass was independently and positively related to the resolution of steatosis.

The underlying mechanism linking skeletal muscle mass and MAFLD has yet to be explored and may include insulin resistance, changes in myokines, chronic inflammation and physical inactivity [38]. It is acknowledged that skeletal muscle is the primary organ responsible for whole-body insulin-mediated glucose utilization, and a reduction in glucose disposal caused by the loss of skeletal muscle mass may further result in insulin resistance [39]. Insulin resistance causes lipolysis of adipose tissue and increases the uptake of free fatty acids, contributing to the accumulation of triglycerides in the liver [40]. On the other hand, insulin resistance leads to skeletal muscle reduction by inhibiting protein synthesis in skeletal muscle through the mammalian target of rapamycin complex 1 (mTORC1) or ribosomal protein S6 kinase beta-1 (S6 K1) pathway and exacerbating proteolytic gluconeogenesis [41–43]. The increase in SMI was significantly associated with the improvement in insulin resistance, which was revealed by a 7-year longitudinal study [27]. Similarly, we also found that an increase in ASM/W was positively correlated with a decrease in HOMA-IR in the correlation analysis and could predict the normalization of HOMA-IR in the ROC analysis. Therefore, elevation of ASM/W may promote the improvement of steatosis by alleviating insulin resistance. However, decreased HOMA-IR was not an independent predictor for the remission of steatosis, suggesting that insulin resistance partly participated in the association of changes in ASM/W and the development or resolution of MAFLD. Myokines such as interleukin-6 and irisin, chronic low-grade inflammation and oxidative stress may also be involved [43].

It has been reported that weight loss is significantly associated with remission of MAFLD [44, 45]. In the present study, the relationships between the increase in ASM/W as well as the decrease in total fat mass and liver steatosis resolution determined by MRI-PDFF were first reported. The logistic regression analysis suggested that an increase in ASM/W is an independent predictor for steatosis improvement. The predicting value of loss of weight for remission of steatosis disappeared in multivariate regression analysis, which may be caused by adjustment of the change in total fat mass. And the main reason for weight loss is decrease in fat mass. In this study, the subgroup analysis based on the changes in weight and ASM/W also provided evidence that an increase in ASM/W is related to a reduction in LFC, regardless of weight loss. In the logistic regression analysis based on whether weight loss, the change of ASM/W was still the independent predictor of improvement of liver steatosis, which demonstrated the positive impact of increase of ASM/W on remission of fatty liver.

To investigate the effect of an increase in ASM/W on hepatic steatosis change in patients with or without obesity, logistic analysis was performed in the subgroups defined by BMI. Decrease in HOMA-IR and a decline in total fat mass independently predict the resolution of liver steatosis in subjects with obesity, suggesting that weight loss may be the primary intervention for these patients. Meanwhile, increasing skeletal muscle mass to improve insulin resistance is also essential. Comparatively, an increase in ASM/W was an independent predictor in participants without obesity, indicating that increasing skeletal muscle mass is the main therapeutic strategy for MAFLD patients without obesity, rather than weight loss. This suggests that increasing skeletal muscle mass can be a promising therapy for MAFLD [46, 47], especially for MAFLD without obesity (BMI<25kg/m^2^). For MAFLD with obesity (BMI≥25kg/m^2^), weight (fat) loss as well as decreasing HOMA-IR are important interventions to improve liver steatosis.

The association between SMI and liver fibrosis has been confirmed in several published studies [12–14, 16, 17] which concluded that low skeletal muscle mass was associated with more severe fibrosis, but all of these findings were cross-sectional. In the current study, we explored the relationship between changes in ASM/W and changes in liver stiffness. Liver stiffness showed a significant decrease at follow-up compared with that at baseline in the group with an ASM/W increase. This result was in accordance with the conclusions reported by previous studies. However, the conclusion needs to be further validated because a small percentage (6, 2.3%) of patients were defined as having liver fibrosis with a baseline LSM > 7.1 kPa, and most of the rest were determined to have no fibrosis with an LSM ≤7.1 kPa in this study. Therefore, further long-term longitudinal research with a large number of subjects with high LSM is needed.

There are some limitations in this study. First, the main limitation of the study is the lack of the assessment of skeletal muscle mass, which can be evaluated by MRI-PDFF, however, it was not available in this study. It will be the purpose in our future studies associated with MAFLD and skeletal muscle. Second, the sample size was small; therefore, the subgroup analysis with a small number of subjects may weaken the reliability of the conclusion. Besides, due the limitations of small sample size regarding orlistat (*n*=16) and meal replacement treatments (*n*=10), subgroup analysis of the associations among different interventions and skeletal muscle mass did not provide sufficient statistical power to demonstrate and further prospective studies with larger sample sizes are needed to identify. Thirdly, this study was implemented in a single center, and more multicenter studies are warranted to provide more powerful evidence. Third, despite the fact that hepatic steatosis remission was evaluated by MRI-PDFF instead of histology, the gold standard, MRI-PDFF was an accurate and invasive technique for assessing the severity of steatosis and could be an alternative method in large-scale population studies.

## Conclusions

In summary, our study suggested that an increase in relative skeletal muscle mass and decrease in total fat mass over time may have a significant beneficial effect on improving the resolution of liver steatosis, independent of weight loss. Increasing skeletal muscle mass might be a strategy for the additive efficacy of MAFLD, especially for patients without obesity. Loss of total fat mass and improvement of insulin resistance are effective strategies for MAFLD with obesity. However, more multicenter longitudinal studies based on large populations are needed to confirm our findings.

### Supplementary information

**Additional file 1: Supplementary figure 1. **Changes in HOMA-IR (A), ALT (B), LFC (C), liver stiffness (D) in the groups classified by changes in ASM and ASM/W. ASM, appendicular skeletal mass; ASM/W, ASM/weight; HOMA-IR, homeostatic model assessment of insulin resistance. Supplementary figure 2. Changes in HOMA-IR (A), ALT (B), LFC (C), liver stiffness (D) in the groups classified by changes in weight and ASM. ASM, appendicular skeletal mass; ASM/W, ASM/weight; HOMA-IR, homeostatic model assessment of insulin resistance. **Supplementary table 1.** Comparison of the baseline and follow-up characteristics in patients from three subgroups with different treatments. **Supplementary table 2.** Comparison of the baseline and follow-up characteristics in patients classified by weight and ASM/W change. **Supplementary table 3.** Comparison of the baseline and follow-up characteristics in patients classified by ASM and ASM/W change. **Supplementary table 4.** Comparison of the baseline and follow-up characteristics in patients classified by weight and ASM change. **Supplementary table 5.** Factors associated with improvement of liver fat content* in univariate logistic regression analysis in all subjects and the subgroup classified by with and without weight loss. **Supplementary table 6.** Comparison of the baseline characteristics in the subgroup classified by BMI <25kg/m2 and BMI≥25kg/m2. **Supplementary table 7.** Factors associated with improvement of liver fat content* in univariate and multivariate logistic regression analysis in subjects classified by BMI <25kg/m2 and BMI≥25kg/m2. **Supplementary table 8.** Factors associated with improvement of liver fat content* in univariate and multivariate logistic regression analysis in subjects classified by BMI <30kg/m2 and BMI≥30kg/m2.

## Data Availability

Some or all datasets generated during and/or analyzed during the current study are not publicly available but are available from the corresponding author on reasonable request.

## Abbreviations

MAFLD: Metabolic associated fatty liver disease
LFC: Liver fat content
ASM: Appendicular skeletal mass
ASM/W: ASM/weight
BMI: Body mass index
MRI-PDFF: Magnetic resonance imaging-based proton density fat fraction
BIA: Bioelectrical impedance analysis
WC: Waist circumference
ALT: Alanine aminotransferase
AST: Aspartate aminotransferase
GGT: γ-glutamyl transpeptidase
ALP: Alkaline phosphatase
FSG: Fasting serum glucose
FIN: Fasting insulin
UA: Uric acid
LSM: Liver stiffness measurement
SMI: Skeletal muscle mass index
ROC: Receiver operating characteristic
AUC: Areas under the curves
TC: Total cholesterol
TG: Triglyceride
HDL-C: High-density lipoprotein cholesterol
LDL-C: Low-density lipoprotein cholesterol
HOMA-IR: Homeostatic model assessment of insulin resistance
WL: Weight loss
ASM/WI: an increase in ASM/W
ASMI: an increase in ASM
