# Supplementary material for: Restoring skeletal muscle mass as an independent determinant of liver fat deposition improvement in MAFLD
Source: Skelet Muscle. 2023 Dec 19;13:23. doi: 10.1186/s13395-023-00333-z (PMC10731792; doi:10.1186/s13395-023-00333-z)
Supplement: Supplementary file 1 — Additional file 1: Supplementary figure 1. Changes in HOMA-IR (A), ALT (B), LFC (C), liver stiffness (D) in the groups classified by changes in ASM and ASM/W. ASM, appendicular skeletal mass; ASM/W, ASM/weight; HOMA-IR, homeostatic model assessment of insulin resistance. Supplementary figure 2. Changes in HOMA-IR (A), ALT (B), LFC (C), liver stiffness (D) in the groups classified by changes in weight and ASM. ASM, appendicular skeletal mass; ASM/W, ASM/weight; HOMA-IR, homeostatic model assessment of insulin resistance. Supplementary table 1. Comparison of the baseline and follow-up characteristics in patients from three subgroups with different treatments. Supplementary table 2. Comparison of the baseline and follow-up characteristics in patients classified by weight and ASM/W change. Supplementary table 3. Comparison of the baseline and follow-up characteristics in patients classified by ASM and ASM/W change. Supplementary table 4. Comparison of the baseline and follow-up characteristics in patients classified by weight and ASM change. Supplementary table 5. Factors associated with improvement of liver fat content* in univariate logistic regression analysis in all subjects and the subgroup classified by with and without weight loss. Supplementary table 6. Comparison of the baseline characteristics in the subgroup classified by BMI <25kg/m2 and BMI≥25kg/m2. Supplementary table 7. Factors associated with improvement of liver fat content* in univariate and multivariate logistic regression analysis in subjects classified by BMI <25kg/m2 and BMI≥25kg/m2. Supplementary table 8. Factors associated with improvement of liver fat content* in univariate and multivariate logistic regression analysis in subjects classified by BMI <30kg/m2 and BMI≥30kg/m2. [file 13395_2023_333_MOESM1_ESM.docx]

**Restoring Skeletal Muscle Mass as An Independent Determinant of Liver Fat Deposition Improvement in MAFLD**

Ting Zhou^a#^, Junzhao Ye^a#^, Ling Luo^a#^, Wei Wang^b^, Shiting Feng^c^, Zhi Dong^c^, Shuyu Zhuo^d*^, Bihui Zhong^a*^

**Affiliations**

^a^ Department of Gastroenterology, The First Affiliated Hospital, Sun Yat-sen University, No. 58 Zhongshan II Road, Yuexiu District, Guangzhou, 510080 China.

^b^ Department of Medical Ultrasonics, Institute of Diagnostic and Interventional Ultrasound, The First Affiliated Hospital, Sun Yat-sen University, No. 58 Zhongshan II Road, Yuexiu District, Guangzhou, Guangdong, 510080 China.

^c^ Department of Radiology, The First Affiliated Hospital, Sun Yat-sen University, No. 58 Zhongshan II Road, Yuexiu District, Guangzhou, Guangdong, 510080 China.

^d^ Department of Nutrition, The First Affiliated Hospital, Sun Yat-sen University, No. 58 Zhongshan II Road, Yuexiu District, Guangzhou, Guangdong, 510080 China.

# These authors contribute equally to this article.

*Corresponding authors:

Shuyu Zhuo, MD, PhD

Department of Nutrition, The First Affiliated Hospital, Sun Yat-sen University, Guangzhou, No. 58 Zhongshan II Road, Yuexiu District, Guangzhou, 510080 China

Phone: (020) 87755766

Email: zhuoshy@mail.sysu.edu.cn

Bihui Zhong, MD, PhD

Department of Gastroenterology, The First Affiliated Hospital, Sun Yat-sen University, No. 58 Zhongshan II Road, Yuexiu District, Guangzhou, 510080 China

Phone: (020) 87755766

Email: zhongbh@mail.sysu.edu.cn


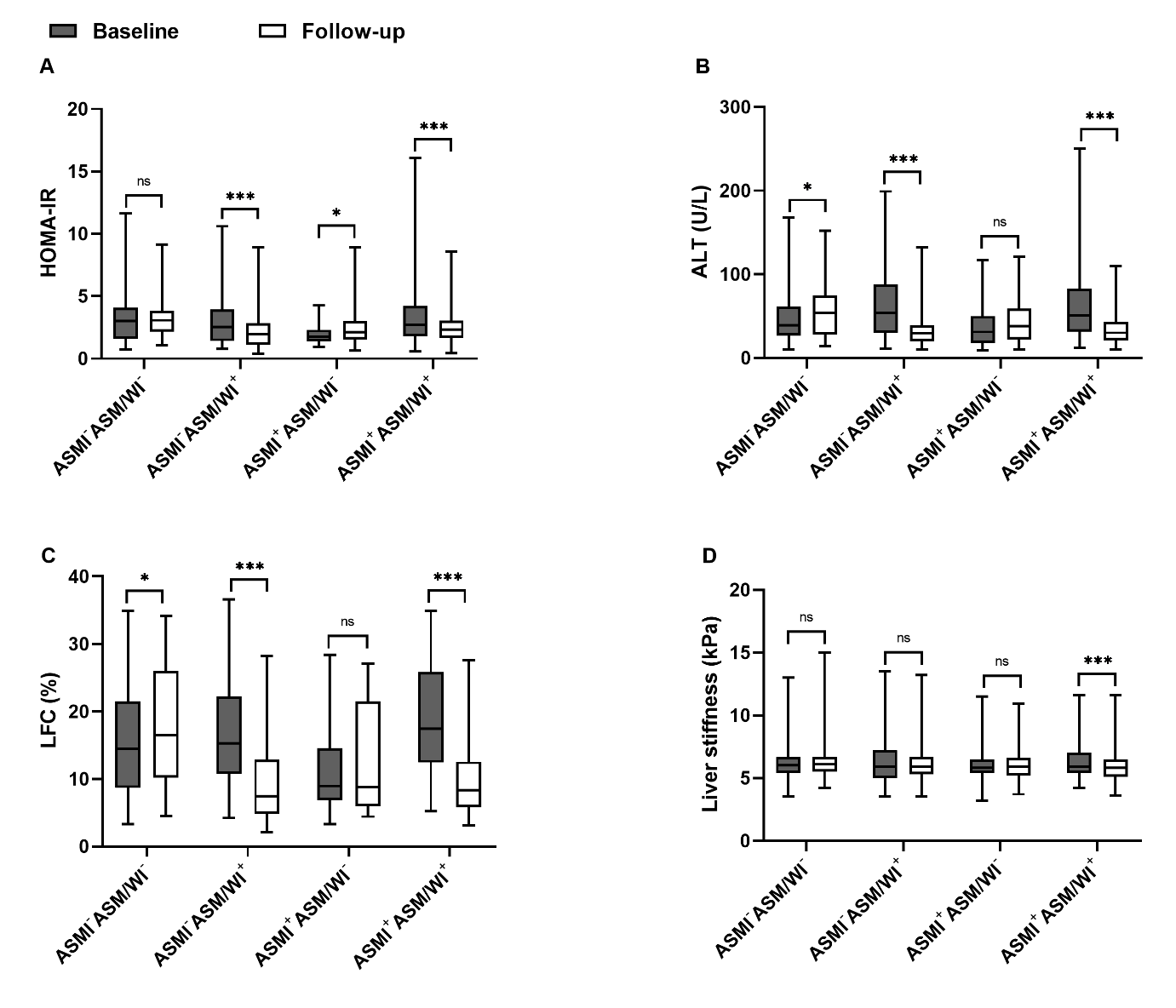
**Supplementary figure 1.** Changes in HOMA-IR (A), ALT (B), LFC (C), liver stiffness (D) in the groups classified by changes in ASM and ASM/W. ASM, appendicular skeletal mass; ASM/W, ASM/weight; HOMA-IR, homeostatic model assessment of insulin resistance.

The ASM/W increase was determined to be △ASM/W>0. △ASM/W =ASM/W _Follow-up-Baseline._ The ASM increase was defined as △ASM>0. △ASM =ASM _Follow-up-Baseline._

ASM/WI^-^, without ASM/W increase; ASM/WI ^+^, with ASM/W increase; ASMI^-^, without ASM increase; ASMI ^+^, with ASM increase. **P*<0.05; ***P*<0.01; ****P*<0.001; ns, not significant.


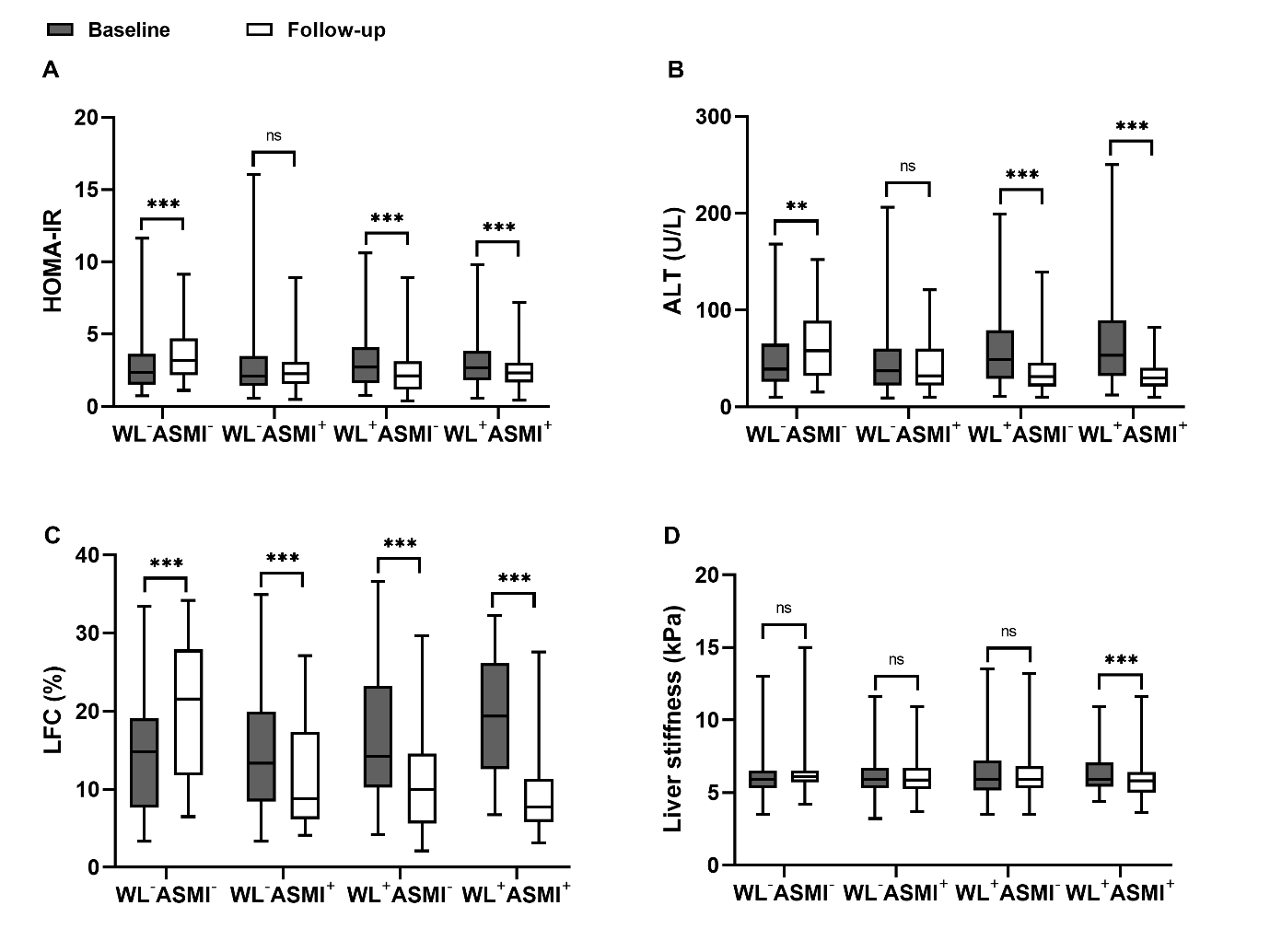


**Supplementary figure 2.** Changes in HOMA-IR (A), ALT (B), LFC (C), liver stiffness (D) in the groups classified by changes in weight and ASM. ASM, appendicular skeletal mass; ASM/W, ASM/weight; HOMA-IR, homeostatic model assessment of insulin resistance.

Weight loss was defined as △weight>0. △weight=weight _Baseline-Follow-up._ The ASM increase was defined as △ASM>0. △ASM =ASM _Follow-up-Baseline._

WL^-^, without weight loss; WL^+^, with weight loss; ASMI^-^, without ASM increase; ASMI ^+^, with ASM increase. **P*<0.05; ***P*<0.01; ****P*<0.001; ns, not significant.

**Supplementary table 1. Comparison of the baseline and follow-up characteristics in patients from three subgroups with different treatments.**

| **Characteristics** | **Orlistat (n=16)** | | |  | **Meal replacement (n=10)** | | | |  | **Lifestyle (n=230)** | | |
| --- | --- | --- | --- | --- | --- | --- | --- | --- | --- | --- | --- | --- |
|  | **Baseline** | **Follow-up** | ***P*** | | **Baseline** | **Follow-up** | | ***P*** | | **Baseline** | **Follow-up** | ***P*** |
| **Weight (kg)** | 84.4±22.9 | 85.6±27.6 | 0.48 | | 73.6±12.8 | | 73.3±12.5 | 0.76 | | 73.3±12.7 | 71.4±12.1 | ＜0.001 |
| **BMI (kg/m^2^)** | 30.2±6.7 | 30.6±8.3 | 0.53 | | 27.4±4.0 | | 27.3±4.2 | 0.72 | | 26.4±3.5 | 25.6±3.3 | ＜0.001 |
| **WC (cm)** | 100.4±11.8 | 99.8±17.6 | 0.77 | | 92.9±8.5 | | 91.7±9.9 | 0.13 | | 91.3±9.1 | 89.2±9.2 | ＜0.001 |
| **ALT (U/L)** | 48 (19, 67) | 54 (32, 77) | 0.33 | | 36 (22, 52) | | 25 (38, 61) | 0.57 | | 48 (29, 76) | 32 (22, 49) | ＜0.001 |
| **AST (U/L)** | 33 (22, 39) | 34 (28, 55) | 0.12 | | 35 (22, 38) | | 28 (21, 46) | 0.84 | | 34 (23, 48) | 26 (21, 36) | ＜0.001 |
| **GGT (U/L)** | 63 (26, 109) | 55 (31, 105) | 0.53 | | 30 (23, 73) | | 37 (29, 56) | 0.54 | | 43 (28, 70) | 33 (25, 47) | ＜0.001 |
| **ALP (U/L)** | 75 (68, 91) | 77 (71, 93) | 0.27 | | 72 (60, 82) | | 75 (60, 86) | 0.08 | | 78 (66, 88) | 76 (66, 91) | 0.43 |
| **TC (mmol/L)** | 4.8±1.1 | 4.9±1.1 | 0.90 | | 4.9±0.7 | | 5.2±0.7 | 0.24 | | 5.0±1.0 | 4.8±0.9 | 0.004 |
| **TG (mmol/L)** | 1.6 (1.2, 2.1) | 1.6 (1.2, 1.8) | 0.49 | | 1.1 (0.8, 1.5) | | 1.2 (1.6, 1.7) | 0.31 | | 1.6 (1.2, 2.2) | 1.5 (1.0, 2.0) | 0.015 |
| **HDL-C (mmol/L)** | 1.2±0.3 | 1.3±0.5 | 0.09 | | 1.2±0.2 | | 1.3±0.2 | 0.12 | | 1.1±0.2 | 1.2±0.4 | 0.017 |
| **LDL-C (mmol/L)** | 2.9±0.9 | 3.2±0.8 | 0.27 | | 3.0±0.5 | | 3.3±0.5 | 0.10 | | 3.1±0.7 | 3.0±0.7 | 0.048 |
| **FSG (mmol/L)** | 4.6±0.6 | 5.3±1.0 | 0.001 | | 5.1±0.7 | | 5.4±0.8 | 0.06 | | 5.2±1.4 | 5.2±1.0 | 0.85 |
| **HOMA-IR** | 2.3 (1.7, 5.1) | 2.2 (1.8, 6.6) | 0.92 | | 3.0 (2.6, 4.9) | | 3.0 (2.2, 3.8) | 0.96 | | 2.5 (1.6, 3.8) | 2.3 (1.5, 3.2) | ＜0.001 |
| **Uric acid (μmol/L)** | 546±190 | 449±162 | 0.06 | | 436±122 | | 404±117 | 0.49 | | 423±96 | 413±91 | 0.14 |
| **Liver stiffness (kPa)** | 6.2 (5.6, 7.5) | 6.0 (5.8, 6.7) | 0.07 | | 5.2 (5.5, 6.2) | | 5.9 (5.2, 6.5) | 0.23 | | 5.9 (5.3, 6.8) | 5.9 (5.2, 6.6) | 0.013 |
| **Liver fat content (%)** | 15.0 (10.3, 22.0) | 10.1 (5.8, 24.5) | 0.046 | | 13.7 (10.2, 16.0) | | 14.6 (7.7, 27.7) | 0.20 | | 14.9 (9.0, 22.7) | 9.0 (5.9, 15.0) | ＜0.001 |
| **ASM (kg)** | 25.8±0.9 | 25.7±8.6 | 0.85 | | 22±4.7 | | 22±4.6 | 0.39 | | 23.8±5.3 | 24.0±5.3 | 0.004 |
| **ASM/W (%)** | 30.1±3.9 | 29.9±4.0 | 0.72 | | 30.2±4.5 | | 30.1±5.2 | 0.86 | | 32.2±4.1 | 33.4±4.3 | ＜0.001 |
| **Body muscle mass** | 27.0±4.9 | 26.8±5.3 | 0.59 | | 24.0±4.6 | | 24.0±4.4 | 0.83 | | 25.9±3.8 | 25.8±3.7 | 0.58 |
| **Total fat mass (kg)** | 30.7±18.0 | 31.2±18.2 | 0.48 | | 24.9±9,5 | | 24.3±10.7 | 0.50 | | 20.2±7.2 | 19.6±7.2 | ＜0.001 |

ASM, appendicular skeletal mass; ASM/W, ASM/weight.

**Supplementary table 2. Comparison of the baseline and follow-up characteristics in patients classified by weight and ASM/W change.**

| **Characteristics** | **Weight loss^-^ASM/W increase^-^ (n=66)** | | |  | **Weight loss^-^ASM/W increase^+^ (n=34)** | | |  | **Weight loss^+^ ASM/W increase^-^ (n=22)** | | |  | **Weight loss^+^ ASM/W increase^+^ (n=134)** | | |
| --- | --- | --- | --- | --- | --- | --- | --- | --- | --- | --- | --- | --- | --- | --- | --- |
|  | **Baseline** | **Follow-up** | ***P*** | | **Baseline** | **Follow-up** | ***P*** | | **Baseline** | **Follow-up** | ***P*** | | **Baseline** | **Follow-up** | ***P*** |
| **Anthropometry** |  |  |  | |  |  |  | |  |  |  | |  |  |  |
| **Weight (kg)** | 72.8±15.6 | 75.5±17.2 | ＜0.001 | | 74.8±14.6  0.66 | 76.1±14.8  0.005 | 0.004 | | 75.4±10.6  0.66 | 74.2±10.6  0.66 | ＜0.001 | | 74.2±13.1 | 69.6±11.8 | ＜0.001 |
| **BMI (kg/m^2^)** | 26.2±4.6 | 27.1±5.2 | ＜0.001 | | 26.3±4.5  0.68 | 26.6±4.4 | 0.001 | | 27.3±3.4  0.68 | 26.7±3.4  0.68 | ＜0.001 | | 26.9±3.4 | 25.2±3.1 | ＜0.001 |
| **WC (cm)** | 92.3±10.8 | 92.7±12.7 | 0.61 | | 92.3±9.7  0.64 | 92.2±9.5 | 0.92 | | 93.0±8.2  0.64 | 91.7±8.8  0.64 | 0.15 | | 91.4±9.0 | 87.8±8.8 | ＜0.001 |
| **Liver biochemistry** |  |  |  | |  |  |  | |  |  |  | |  |  |  |
| **ALT (U/L)** | 32 (20, 56) | 43 (28, 77) | ＜0.001 | | 47 (31, 74)  0.17 | 29 (21, 61)  ＜0.001 | 0.003 | | 39 (27, 58)  0.17 | 41 (22, 70)  0.17 | 0.95 | | 54(32, 89) | 30(21, 39) | ＜0.001 |
| **AST (U/L)** | 28 (21, 39) | 32 (22, 43) | 0.001 | | 34 (25, 55)  0.07 | 27 (21, 32)  ＜0.001 | ＜0.001 | | 28 (22, 38)  0.07 | 31 (20, 40)  0.07 | 0.49 | | 37(27, 51) | 25(21, 34) | ＜0.001 |
| **GGT (U/L)** | 33 (24, 58) | 43 (26, 61) | ＜0.001 | | 47 (28, 85)  0.23 | 31 (25, 45)  ＜0.001 | 0.001 | | 33 (26, 44)  0.23 | 34 (25, 57)  0.23 | 0.99 | | 49(45, 77) | 31(23, 44) | ＜0.001 |
| **ALP (U/L)** | 73 (64, 86) | 77 (66, 91) | 0.045 | | 73 (54, 89)  0.61 | 72 (55, 92)  0.32 | 0.47 | | 81 (62, 85)  0.61 | 76 (65, 84)  0.61 | 0.29 | | 80(68, 89) | 75(67, 91) | 0.70 |
| **Metabolic characteristics** |  |  |  | |  |  |  | |  |  |  | |  |  |  |
| **TC (mmol/L)** | 5.1±0.8 | 5.0±0.8 | 0.67 | | 4.9±1.1  0.15 | 4.8±0.8  0.011 | 0.37 | | 5.0±1.0  0.15 | 4.9±0.9  0.15 | 0.76 | | 5.0±1.0 | 4.8±0.9 | 0.008 |
| **TG (mmol/L)** | 1.4 (1.2, 2.9) | 1.6 (1.1, 2.0) | 0.37 | | 1.5 (1.1, 2.3)  0.62 | 1.3 (1.2, 2.0)  0.008 | 0.37 | | 1.7 (1.3, 2.2)  0.62 | 1.9 (1.3, 2.4)  0.62 | 0.54 | | 1.6 (1.2, 2.4) | 1.4(0.9, 2.0) | 0.001 |
| **HDL-C (mmol/L)** | 1.2±0.2 | 1.2±0.3 | 0.90 | | 1.2±0.3  0.46 | 1.2±0.3  0.039 | 0.62 | | 1.1±0.2  0.46 | 1.2±0.5  0.46 | 0.33 | | 1.1±0.2 | 1.2±0.4 | 0.004 |
| **LDL-C (mmol/L)** | 3.1±0.7 | 3.2±0.6 | 0.30 | | 3.0±0.9  0.86 | 3.0±0.7  0.010 | 0.62 | | 3.1±0.8  0.86 | 3.1±0.8  0.86 | 0.94 | | 3.2±0.7 | 3.0±0.7 | 0.011 |
| **FSG (mmol/L)** | 4.9±0.8 | 5.4±1.0 | ＜0.001 | | 5.1±1.2  0.74 | 4.9±0.9  0.92 | 0.17 | | 6.0±1.4  0.74 | 6.1±1.5  0.74 | 0.70 | | 5.2±1.5 | 5.1±0.9 | 0.24 |
| **HOMA-IR** | 1.9 (1.5, 2.9) | 2.6 (1.8, 3.5) | ＜0.001 | | 2.8 (1.8, 4.4)  0.96 | 2.4 (1.6, 3.3)  0.05 | 0.001 | | 3.2 (2.3, 5.1)  0.96 | 2.9 (2.1, 3.5)  0.96 | 0.03 | | 2.7 (1.7, 3.9) | 2.1(1.4, 3.0) | ＜0.001 |
| **Uric acid (μmol/L)** | 447±119 | 432±84 | 0.34 | | 425±113  0.86 | 422±122  0.11 | 0.80 | | 415±76  0.86 | 474±102  0.86 | 0.014 | | 428±109 | 395±92.9 | ＜0.001 |
| **Liver stiffness (kPa)** | 5.9 (5.3, 6.5) | 6.0 (5.3, 6.6) | 0.66 | | 6.0 (5.3, 6.9)  0.55 | 5.8 (5.3, 6.8)  0.77 | 0.45 | | 6.3 (5.6, 7.5)  0.55 | 6.1 (5.4, 7.4)  0.55 | 0.53 | | 5.9(5.3, 7.2) | 5.8(5.2, 6.5) | 0.001 |
| **Liver fat content (%)** | 11.4 (7.4, 18.4) | 15.1 (7.0, 25.3) | 0.008 | | 16.9 (10.3, 26.0)  0.002 | 8.8 (6.6, 14.7)  ＜0.001 | ＜0.001 | | 13.3 (8.8, 24.5)  0.002 | 13.8 (9.0, 20.3)  0.002 | 0.30 | | 17.5(11.2, 24.8) | 7.7(5.5, 12.0) | ＜0.001 |
| **Body composition** |  |  |  | |  |  |  | |  |  |  | |  |  |  |
| **ASM (kg)** | 23.6±5.9 | 23.7±60 | 0.10 | | 24.5±5.4  0.83 | 25.6±5.8 | ＜0.001 | | 24.4±4.4  0.83 | 23.6±4.3  0.83 | ＜0.001 | | 23.7±5.7 | 23.8±5.4 | 0.18 |
| **ASM/W (%)** | 32.4±4.3 | 31.4±4.5 | ＜0.001 | | 32.8±3.3  0.98 | 33.5±3.6 | ＜0.001 | | 32.4±4.2  0.98 | 31.9±4.3  0.98 | ＜0.001 | | 31.6±4.1 | 33.9±4.4 | ＜0.001 |
| **Body muscle mass (kg)** | 25.4±4.2 | 25.6±4.2 | 0.27 | | 26.6±3.5 | 26.7±3.3 | 0.65 | | 26.7±3.5 | 26.8±3.4 | 0.71 | | 25.8±4.0 | 25.7±4.0 | 0.038 |
| **Total fat mass (kg)** | 21.9±11.3 | 22.9±11.4 | ＜0.001 | | 21.0±10.3 | 21.2±10.6 | 0.39 | | 21.4±8.2 | 20.9±8.3 | 0.20 | | 20.6±6.9 | 19.1±6.7 | ＜0.001 |

Continuous variables are reported as mean ± standard deviation (SD) or median (interquartile range). MAFLD, metabolic associated fatty liver disease; BMI, body mass index; WC, Waist circumference； ALT, Alanine aminotransferase; AST, Aspartate aminotransferase, GGT, γ-glutamyl transpeptidase; ALP, alkaline phosphatase; TC, total cholesterol; TG, triglyceride; HDL-C, high-density lipoprotein cholesterol; LDL-C, low-density lipoprotein cholesterol; FSG, fasting serum glucose; HOMA-IR, homeostatic model assessment of insulin resistance; ASM, appendicular skeletal mass; ASM/W, ASM/weight. Weight loss^-^, △Weight _Baseline-Follow-up_ ≤0; Weight loss^+^, △Weight _Baseline-Follow-up_ >0; ASM/W increase^-^,△ASM/W _Follow-up-Baseline_ ≤0; ASM/W increase^-^, △ASM/W _Follow-up-Baseline_ >0.

**Supplementary table 3. Comparison of the baseline and follow-up characteristics in patients classified by ASM and ASM/W change.**

| **Characteristics** | **ASM increase^-^ASM/W increase^-^ (n=53)** | | |  | **ASM increase ^-^ASM/W increase^+^ (n=68)** | | |  | **ASM increase ^+^ ASM/W increase^-^ (n=35)** | | |  | **ASM increase ^+^ ASM/W increase^+^ (n=100)** | | |
| --- | --- | --- | --- | --- | --- | --- | --- | --- | --- | --- | --- | --- | --- | --- | --- |
|  | **Baseline** | **Follow-up** | ***P*** | | **Baseline** | **Follow-up** | ***P*** | | **Baseline** | **Follow-up** | ***P*** | | **Baseline** | **Follow-up** | ***P*** |
| **Anthropometry** |  |  |  | |  |  |  | |  |  |  | |  |  |  |
| **Weight (kg)** | 75.4±15.9 | 76.7±17.8 | 0.014 | | 75.9±14.0  0.66 | 70.1±12.3  0.005 | ＜0.001 | | 70.4±11.6  0.66 | 72.8±11.8  0.66 | ＜0.001 | | 73.3±12.8 | 71.5±13.0 | ＜0.001 |
| **BMI (kg/m^2^)** | 27.3±4.6 | 27.7±5.4 | 0.032 | | 27.1±3.8  0.68 | 25.2±3.5 | ＜0.001 | | 25.3±3.6  0.68 | 26.1±3.7  0.68 | ＜0.001 | | 26.5±3.5 | 25.7±3.4 | ＜0.001 |
| **WC (cm)** | 93.7±10.6 | 93.5±12.9 | 0.74 | | 92.1±9.6  0.64 | 88.1±9.6 | ＜0.001 | | 90.6±9.2  0.64 | 90.8±9.7  0.64 | 0.80 | | 91.3±8.9 | 89.1±8.7 | ＜0.001 |
| **Liver biochemistry** |  |  |  | |  |  |  | |  |  |  | |  |  |  |
| **ALT (U/L)** | 39 (27, 62) | 54 (28, 75) | 0.014 | | 54 (30, 88)  0.17 | 30 (20, 39)  ＜0.001 | ＜0.001 | | 31 (18, 50)  0.17 | 38 (22, 59)  0.17 | 0.08 | | 51(31, 83) | 30(21, 43) | ＜0.001 |
| **AST (U/L)** | 32 (21, 38) | 37 (23, 54) | 0.001 | | 37 (27, 49)  0.07 | 24 (21, 35)  ＜0.001 | ＜0.001 | | 25 (21, 35)  0.07 | 30 (22, 40)  0.07 | 0.31 | | 37(26, 55) | 26(21, 32) | ＜0.001 |
| **GGT (U/L)** | 34 (25, 62) | 40 (26, 64) | 0.018 | | 49 (32, 81)  0.23 | 34 (24, 43)  ＜0.001 | ＜0.001 | | 30 (24, 46)  0.23 | 37 (25, 54)  0.23 | 0.003 | | 48(32, 72) | 31(23, 45) | ＜0.001 |
| **ALP (U/L)** | 77 (63, 87) | 79 (64, 93) | 0.008 | | 82 (67, 89)  0.61 | 78 (65, 92)  0.32 | 0.38 | | 71 (65, 82)  0.61 | 77 (69, 82)  0.61 | 0.84 | | 78(66, 89) | 73(66, 91) | 0.50 |
| **Metabolic characteristics** |  |  |  | |  |  |  | |  |  |  | |  |  |  |
| **TC (mmol/L)** | 5.1±0.9 | 5.0±0.8 | 0.47 | | 5.0±1.0  0.15 | 4.7±0.9  0.011 | 0.025 | | 4.9±0.7  0.15 | 4.9±0.9  0.15 | 0.80 | | 5.0±1.0 | 4.8±0.9 | 0.08 |
| **TG (mmol/L)** | 1.5 (1.2, 2.1) | 1.7 (1.3, 2.1) | 0.20 | | 1.6 (1.0, 2.3)  0.62 | 1.4 (0.9, 1.9)  0.008 | 0.008 | | 1.4 (1.2, 2.2)  0.62 | 1.5 (1.0, 1.9)  0.62 | 0.91 | | 1.5 (1.2, 2.3) | 1.4(1.1, 2.0) | 0.02 |
| **HDL-C (mmol/L)** | 1.2±0.2 | 1.2±0.4 | 0.14 | | 1.1±0.2  0.46 | 1.2±0.3  20.039 | 0.013 | | 1.1±03  0.46 | 1.1±0.4  0.46 | 0.58 | | 1.2±0.3 | 1.2±0.4 | 0.036 |
| **LDL-C (mmol/L)** | 3.2±0.7 | 3.2±0.6 | 0.81 | | 3.2±0.7  0.86 | 3.0±0.7  0.010 | 0.02 | | 3.1±0.7  0.86 | 3.2±0.7  0.86 | 0.19 | | 3.1±0.8 | 3.0±0.7 | 0.19 |
| **FSG (mmol/L)** | 5.4±1.2 | 5.8±1.3 | 0.02 | | 5.2±1.0  0.74 | 5.2±0.9  0.92 | 0.54 | | 4.8±0.7  0.74 | 5.1±0.9  0.74 | 0.001 | | 5.3±1.7 | 5.0±0.9 | 0.15 |
| **HOMA-IR** | 3.0 (1.6, 4.1) | 3.1 (2.2, 3.8) | 0.15 | | 2.5 (1.4, 4.0)  0.96 | 1.9 (1.1, 2.8)  0.05 | ＜0.001 | | 1.7 (1.4, 2.3)  0.96 | 2.1 (1.5, 3.0)  0.96 | 0.032 | | 2.7 (1.8, 4.2) | 2.3(1.7, 3.0) | ＜0.001 |
| **Uric acid (μmol/L)** | 454±123 | 450±95 | 0.84 | | 446±121  0.86 | 397±96  0.11 | ＜0.001 | | 416±83  0.86 | 431±83  0.86 | 0.32 | | 414±99 | 403±103 | 0.24 |
| **Liver stiffness (kPa)** | 6.0 (5.4, 6.7) | 6.1 (5.5, 6.6) | 0.39 | | 5.9 (5.0, 7.2)  0.55 | 5.9 (5.3, 6.7)  0.77 | 0.44 | | 5.8 (5.4, 6.5)  0.55 | 5.9 (5.2, 6.6)  0.55 | 0.53 | | 5.9(5.4, 7.0) | 5.8(5.1, 6.5) | ＜0.001 |
| **Liver fat content (%)** | 14.5 (8.7, 21.5) | 26.0 (10.2, 16.5) | 0.019 | | 15.3 (10.8, 22.2)  0.002 | 7.4 (4.8, 12.9)  ＜0.001 | ＜0.001 | | 8.9 (6.9, 14.5)  0.002 | 8.8 (6.0, 21.5)  0.55 | 0.96 | | 17.4(12.4, 25.9) | 8.3(5.8, 12.6) | ＜0.001 |
| **Body composition** |  |  |  | |  |  |  | |  |  |  | |  |  |  |
| **ASM (kg)** | 24.4±5.8 | 23.9±5.8 | ＜0.001 | | 24.8±5.5  0.83 | 24.3±5.3 | ＜0.001 | | 22.9±5.1  0.83 | 23.5±5.3  0.83 | ＜0.001 | | 23.3±5.6 | 24.2±5.8 | ＜0.001 |
| **ASM/W (%)** | 32.2±3.7 | 31.1±3.9 | ＜0.001 | | 32.5±3.8  0.98 | 34.5±4.0 | ＜0.001 | | 32.5±5.0  0.98 | 32.2±5.0  0.98 | 0.007 | | 31.4±4.1 | 33.4±4.4 | ＜0.001 |
| **Body muscle mass (kg)** | 26.0±4.1 | 26.2±4.1 | 0.17 | | 26.3±3.7 | 26.1±3.6 | 0.17 | | 25.4±4.1 | 25.4±3.9 | 0.95 | | 25.8±4.0 | 25.7±4.0 | 0.37 |
| **Total fat mass (kg)** | 23.3±11.6 | 23.6±11.8 | 0.17 | | 20.5±7.6 | 18.1±6.9 | ＜0.001 | | 19.5±8.3 | 20.6±8.7 | ＜0.001 | | 20.8±7.7 | 20.5±8.0 | 0.06 |

Continuous variables are reported as mean ± standard deviation (SD) or median (interquartile range). MAFLD, metabolic associated fatty liver disease; BMI, body mass index; WC, Waist circumference； ALT, Alanine aminotransferase; AST, Aspartate aminotransferase, GGT, γ-glutamyl transpeptidase; ALP, alkaline phosphatase; TC, total cholesterol; TG, triglyceride; HDL-C, high-density lipoprotein cholesterol; LDL-C, low-density lipoprotein cholesterol; FSG, fasting serum glucose; HOMA-IR, homeostatic model assessment of insulin resistance; ASM, appendicular skeletal mass; ASM/W, ASM/weight. ASM increase^-^,△ASM _Follow-up-Baseline_ ≤0; ASM increase^-^, △ASM _Follow-up-Baseline_ >0; ASM/W increase^-^,△ASM/W _Follow-up-Baseline_ ≤0; ASM/W increase^-^, △ASM/W _Follow-up-Baseline_ >0.

| **Characteristics** | **Weight loss^-^ASM increase^-^ (n=31)** | | |  | **Weight loss^-^ASM increase^+^ (n=69)** | | |  | **Weight loss^+^ ASM increase^-^ (n=90)** | | |  | **Weight loss^+^ ASM increase^+^ (n=134)** | | |
| --- | --- | --- | --- | --- | --- | --- | --- | --- | --- | --- | --- | --- | --- | --- | --- |
|  | **Baseline** | **Follow-up** | ***P*** | | **Baseline** | **Follow-up** | ***P*** | | **Baseline** | **Follow-up** | ***P*** | | **Baseline** | **Follow-up** | ***P*** |
| **Anthropometry** |  |  |  | |  |  |  | |  |  |  | |  |  |  |
| **Weight (kg)** | 75.5±18.9 | 78.5±21.5 | ＜0.001 | | 72.6±13.2  0.66 | 74.4±13.4  0.005 | ＜0.001 | | 75.8±13.2  0.66 | 71.1±12.0  0.66 | ＜0.001 | | 72.6±11.9 | 69.2±11.3 | ＜0.001 |
| **BMI (kg/m^2^)** | 27.3±5.4 | 28.4±6.4 | ＜0.001 | | 25.8±4.1  0.68 | 26.3±4.1 | ＜0.001 | | 27.2±3.7  0.68 | 25.4±3.5  0.68 | ＜0.001 | | 26.6±2.9 | 25.3±2.7 | ＜0.001 |
| **WC (cm)** | 94.2±12.2 | 94.7±15.2 | 0.66 | | 91.4±9.4  0.64 | 91.5±9.6 | 0.88 | | 92.3±9.2  0.64 | 89.0±9.4  0.64 | ＜0.001 | | 90.8±8.5 | 87.5±7.9 | ＜0.001 |
| **Liver biochemistry** |  |  |  | |  |  |  | |  |  |  | |  |  |  |
| **ALT (U/L)** | 39 (26, 65) | 58 (32, 89) | 0.004 | | 37 (22, 60)  0.17 | 32 (22, 60)  ＜0.001 | 0.28 | | 49 (29, 79)  0.17 | 31 (21, 46)  0.17 | ＜0.001 | | 53(32, 90) | 30(21, 41) | ＜0.001 |
| **AST (U/L)** | 34 (20, 39) | 37 (23, 54) | 0.001 | | 30 (21, 39)  37 (27, 49)  0.07 | 27 (22, 36)  ＜0.001 | 0.025 | | 35 (24, 46)  0.07 | 25 (21, 36)  0.07 | ＜0.001 | | 37(27, 55) | 25(20, 34) | ＜0.001 |
| **GGT (U/L)** | 36 (25, 82) | 41 (24, 63) | 0.008 | | 40 (24, 62)  0.23 | 34 (25, 51)  ＜0.001 | 0.66 | | 43 (29, 77)  0.23 | 34 (24, 44)  0.23 | ＜0.001 | | 48(35, 66) | 30(22, 45) | ＜0.001 |
| **ALP (U/L)** | 75 (64, 92) | 82 (63, 96) | 0.011 | | 71 (62, 83)  0.61 | 77 (62, 87)  0.32 | 0.78 | | 81 (66, 88)  0.61 | 78 (65, 90)  0.61 | 0.23 | | 78(69, 89) | 74(70, 91) | 0.71 |
| **Metabolic characteristics** |  |  |  | |  |  |  | |  |  |  | |  |  |  |
| **TC (mmol/L)** | 5.2±0.9 | 5.1±0.7 | 0.48 | | 4.9±0.9  0.15 | 4.8±0.9  0.011 | 0.55 | | 5.0±1.0  0.15 | 4.8±0.9  0.15 | 0.06 | | 5.0±1.0 | 4.8±1.0 | 0.13 |
| **TG (mmol/L)** | 1.4 (1.2, 1.9) | 1.7 (1.3, 2.0) | 0.28 | | 1.4 (1.2, 2.2)  0.62 | 1.4 (1.1, 1.9)  0.008 | 0.64 | | 1.7 (1.1, 2.2)  0.62 | 1.6 (1.0, 2.0)  0.62 | 0.0 | | 1.6 (1.2, 2.4) | 1.5(0.9, 2.0) | 0.031 |
| **HDL-C (mmol/L)** | 1.2±0.2 | 1.2±0.3 | 0.20 | | 1.1±0.3  0.46 | 1.1±0.3  20.039 | 0.76 | | 1.1±0.2  0.46 | 1.2±0.3  0.46 | 0.017 | | 1.2±0.3 | 1.3±0.5 | 0.042 |
| **LDL-C (mmol/L)** | 3.2±0.7 | 3.3±0.5 | 0.77 | | 3.0±0.8  0.86 | 3.1±0.7  0.010 | 0.72 | | 3.1±0.7  0.86 | 3.0±0.7  0.86 | 0.11 | | 3.2±0.7 | 3.0±0.7 | 0.20 |
| **FSG (mmol/L)** | 5.0±0.9 | 5.7±1.1 | ＜0.001 | | 5.0±1.0  0.74 | 5.0±0.9  0.92 | 0.47 | | 5.4±1.2  0.74 | 5.3±1.2  0.74 | 0.96 | | 5.3±1.9 | 5.0±0.9 | 0.32 |
| **HOMA-IR** | 2.4 (1.5, 3.6) | 3.2 (2.2, 4.7) | ＜0.001 | | 2.1 (1.4, 3.5)  0.96 | 2.3 (1.6, 3.1)  0.05 | 0.33 | | 2.7 (1.6, 4.1)  0.96 | 2.1 (1.2, 3.1)  0.96 | ＜0.001 | | 2.7 (1.8, 3.8) | 2.3(1.7, 3.0) | ＜0.001 |
| **Uric acid (μmol/L)** | 483±143 | 433±87 | 0.10 | | 420±98  0.86 | 426±103  0.11 | 0.58 | | 439±113  0.86 | 416±102  0.86 | 0.06 | | 407±90 | 393±90 | 0.21 |
| **Liver stiffness (kPa)** | 5.9 (5.3, 6.5) | 6.1 (5.7, 6.5) | 0.12 | | 5.9 (5.3, 6.7)  0.55 | 5.9 (5.2, 6.7)  0.77 | 0.34 | | 5.9 (5.2, 7.2)  0.55 | 5.9 (5.3, 6.8)  0.55 | 0.37 | | 5.9(5.4, 7.1) | 5.8(5.0, 6.4) | ＜0.001 |
| **Liver fat content (%)** | 14.8 (7.7, 19.1) | 21.6 (11.8, 27.9) | ＜0.001 | | 13.3 (8.4, 20.0)  0.002 | 8.8 (6.2, 17.4)  ＜0.001 | ＜0.001 | | 14.2 (10.2, 23.2)  0.002 | 10.0 (5.6, 14.6)  0.55 | ＜0.001 | | 19.4(12.6, 26.2) | 7.7(5.8, 11.3) | ＜0.001 |
| **Body composition** |  |  |  | |  |  |  | |  |  |  | |  |  |  |
| **ASM (kg)** | 24.4±6.8 | 24.1±6.7 | 0.008 | | 23.7±5.3  0.83 | 24.5±5.6 | ＜0.001 | | 24.7±5.3  0.83 | 24.1±5.0  0.83 | ＜0.001 | | 22.6±5.7 | 23.3±5.7 | ＜0.001 |
| **ASM/W (%)** | 32.1±3.4 | 30.5±3.6 | ＜0.001 | | 32.6±4.2  0.98 | 32.8±4.4 | 0.059 | | 32.4±3.9  0.98 | 33.8±4.2  0.98 | ＜0.001 | | 30.7±4.3 | 33.3±4.7 | ＜0.001 |
| **Body muscle mass (kg)** | 25.5±4.4 | 25.7±4.5 | 0.15 | | 26.0±3.8 | 26.1±3.6 | 0.69 | | 26.4±3.6 | 26.3±3.6 | 0.29 | | 25.4±4.2 | 25.2±4.2 | 0.11 |
| **Total fat mass (kg)** | 24.7±13.6 | 25.5±13.6 | 0.002 | | 20.2±9.3 | 20.9±9.6 | 0.001 | | 20.7±7.7 | 18.8±7.3 | ＜0.001 | | 20.8±6.2 | 20.2±6.4 | 0.002 |

**Supplementary table 4.** **Comparison of the baseline and follow-up characteristics in patients classified by weight and ASM change.**

Continuous variables are reported as mean ± standard deviation (SD) or median (interquartile range). MAFLD, metabolic associated fatty liver disease; BMI, body mass index; WC, Waist circumference； ALT, Alanine aminotransferase; AST, Aspartate aminotransferase, GGT, γ-glutamyl transpeptidase; ALP, alkaline phosphatase; TC, total cholesterol; TG, triglyceride; HDL-C, high-density lipoprotein cholesterol; LDL-C, low-density lipoprotein cholesterol; FSG, fasting serum glucose; HOMA-IR, homeostatic model assessment of insulin resistance; ASM, appendicular skeletal mass; ASM/W, ASM/weight. Weight loss^-^, △Weight _Baseline-Follow-up_ ≤0; Weight loss^+^, △Weight _Baseline-Follow-up_ >0; ASM/W increase^-^,△ASM/W _Follow-up-Baseline_ ≤0; ASM/W increase^-^, △ASM/W _Follow-up-Baseline_ >0.

**Supplementary table 5.** **Factors associated with improvement of liver fat content* in univariate logistic regression analysis in all subjects and the subgroup classified by with and without weight loss.**

| **Factors** | **All subjects** | | |  | **Without weight loss ^#^** | |  | **With weight loss ^#^** | |
| --- | --- | --- | --- | --- | --- | --- | --- | --- | --- |
|  | **Univariate** | | **Univariate** | | | | | **Univariate** | |
|  | **OR (95% CI)** | ***P*** | **OR (95% CI)** | | | ***P*** | | **OR (95% CI)** | ***P*** |
| **Age, years** | 1.00 (1.01-1.03) | 0.42 | 1.01(0.99-1.05) | | | 0.34 | | 1.00(0.98-1.03) | 0.83 |
| **Male** | 0.79 (0.45-1.39) | 0.41 | 1.10(0.42-2.88) | | | 0.85 | | 0.55(0.23-1.32) | 0.18 |
| **Hypertension** | 1.04 (0.61-1.76) | 0.89 | 1.54(0.65-3.67) | | | 0.33 | | 1.01(0.46-2.25) | 0.97 |
| **Diabetes** | 1.41 (0.70-2.87) | 0.34 | 2.21(0.68-7.26) | | | 0.19 | | 0.92(0.36-2.39) | 0.87 |
| **Smoke** | 1.05 (0.48-2.31) | 0.91 | 0.94(0.23-3.93) | | | 0.93 | | 0.88(0.29-2.67) | 0.83 |
| **Alcohol consumption** | 0.82 (0.32-2.11) | 0.68 | 0.28(0.03-2.32) | | | 0.24 | | 0.62(0.20-1.95) | 0.41 |
| **Weight (kg)** | 0.99 (0.97-1.01) | 0.25 | 0.99(0.96-1.02) | | | 0.53 | | 0.98(0.95-1.01) | 0.20 |
| **BMI, kg/m2** | 1.13(0.67-1.91) | 0.65 | 0.98(0.89-1.08) | | | 0.66 | | 0.93(0.84-1.04) | 0.20 |
| **WC, cm** | 0.98(0.92-1.05) | 0.52 | 0.98(0.94-1.03) | | | 0.45 | | 0.97(0.93-1.01) | 0.13 |
| **Insulin resistance** | 1.51(0.90-2.55) | 0.12 | 1.53(0.63-3.72) | | | 0.35 | | 1.05(0.48-2.29) | 0.90 |
| **ASM/W (%)** | 0.96 (0.90-1.02) | 0.21 | 0.99(0.89-1.10) | | | 0.84 | | 0.97(0.89-1.06) | 0.53 |
| **Body muscle mass (kg)** | 0.96(0.90-1.03) | 0.25 | 0.99(0.90-1.11) | | | 0.93 | | 0.93(0.85-1.03) | 0.15 |
| **Total fat mass (kg)** | 0.98 (0.95-1.01) | 0.16 | 0.98(0.94-1.03) | | | 0.40 | | 0.98(0.93-1.03) | 0.45 |
| **Decrease in Weight ^a^ (kg)** | 1.49 (1.33-1.67) | <0.001 | - | | | - | | - | - |
| **Decrease in HOMA-IR ^b^** | 3.65 (2.12-6.26) | <0.001 | 3.00(1.24-7.27)  27) | | | 0.015 | | 2.28(1.02-5.08) | 0.044 |
| **Intervention** |  |  |  | | |  | |  |  |
| **Orlistat** | 1 |  | 1 | | |  | | 1 |  |
| **Meal replacement** | 1.11(0.22-5.62) | 0.90 | 0.75(0.06-9.62) | | | 0.83 | | 0.50(0.03-8.96) | 0.64 |
| **Lifestyle** | 2.44(0.86-6.94) | 0.10 | 1.37(0.34-5.48) | | | 0.66 | | 1.05(1.06-10.42) | 0.97 |
| **△ASM/W _Baseline-Follow-up_ (%)** | 2.34 (1.83-2.99) | <0.001 | 2.78(1.49-5.21) | | | 0.001 | | 1.91(1.40-2.62) | <0.001 |
| △**Body muscle mass _Baseline -Follow-up_ (kg)** | 0.91(0.71-1.16) | 0.44 | 0.28(1.30-2.08) | | | 0.28 | | 0.84(0.59-1.20) | 0.34 |
| △**Total fat mass _Baseline -Follow-up_ (kg)** | 1.39 (1.13-1.72) | 0.002 | 2.85(1.81-4.48) | | | <0.001 | | 3.29(2.24-4.82) | <0.001 |

OR, odds ratio; CI, confidence interval; WC, Waist circumference; HOMA-IR, homeostatic model assessment of insulin resistance; ASM, appendicular skeletal mass; ASM/W, ASM/weight.

^#^Weight loss, △Weight _Baseline-Follow-up_ >0; ^a^ Decrease in Weight was defined as △Weight _Baseline-Follow-up_ >0; ^b^ Decrease in HOMA-IR was defined as △HOMA-IR _Baseline-Follow-up_ >0. *The improvement of liver steatosis was defined as the liver fat content (LFC) ≥30% relative decline compared with baseline in MRI-PDFF (LFC _Baseline-Follow-up_/ LFC _Baseline_≥30%).

**Supplementary table 6. Comparison of the baseline characteristics in the subgroup classified by BMI <25kg/m^2^ and BMI≥25kg/m^2^..**

| **Characteristics** | **BMI <25kg/m^2^** | | |  | **BMI ≥25kg/m^2^** | | |  |
| --- | --- | --- | --- | --- | --- | --- | --- | --- |
|  | **With ASM/W increase (n=57)** | **Without ASM/W increase (n=33)** | ***P*** |  | **With ASM/W increase (n=111)** | **Without ASM/W increase (n=55)** | ***P*** |  |
|  |  |  |  |  |  |  |  |  |
| **Demographic** |  |  |  |  |  |  |  |  |
| **Age(years)** | 44.3±14.9 | 41.1±13.0 | 0.32 |  | 39.2±14.9 | 41.6±12.9 | 0.32 |  |
| **Male, n (%)** | 37 (64.9) | 21 (63.6) | 0.39 |  | 85 (76.6) | 39 (70.9) | 0.43 |  |
| **Anthropometric** |  |  |  |  |  |  |  |  |
| **Weight (kg)** | 63.2±6.9 | 61.9±6.7 | 0.39 |  | 80.1±12.1 | 80.4±13.5 | 0.90 |  |
| **BMI (kg/m^2^)** | 23.2±1.2 | 22.9±1.2 | 0.22 |  | 28.6±3.0 | 28.7±4.1 | 0.88 |  |
| **WC (cm)** | 83.5±4.9 | 83.5±5.1 | 0.99 |  | 95.7±8.0 | 97.9±8.5 | 0.12 |  |
| **Diastolic blood pressure (mmHg)** | 129±18 | 131±15 | 0.49 |  | 130±16 | 128±17 | 0.65 |  |
| **Systolic blood pressure (mmHg)** | 84±11 | 87±15 | 0.22 |  | 86±12 | 83±11 | 0.14 |  |
| **Liver biochemistry** |  |  |  |  |  |  |  |  |
| **ALT (U/L)** | 47 (30, 93) | 34 (18, 50) | 0.019 |  | 54 (32, 81) | 38 (25, 65) | 0.003 |  |
| **AST (U/L)** | 36 (28, 47) | 28 (21, 35) | 0.001 |  | 37 (26, 52) | 32 (21, 39) | 0.002 |  |
| **GGT (U/L)** | 46 (34, 81) | 32 (24, 44) | 0.008 |  | 50 (32, 77) | 35 (25, 60) | 0.008 |  |
| **ALP (U/L)** | 78 (72, 88) | 73 (60, 87) | 0.37 |  | 78 (66, 91) | 74 (65, 85) | 0.18 |  |
| **Metabolic characteristics** |  |  |  |  |  |  |  |  |
| **TC (mmol/L)** | 5.2±0.9 | 5.0±0.8 | 0.33 |  | 4.9±1.1 | 5.1±0.9 | 0.32 |  |
| **TG (mmol/L)** | 1.8 (1.2, 2.3) | 1.2 (1.0, 1.6) | 0.005 |  | 1.5 (1.0, 2.4) | 1.6 (1.2, 2.1) | 0.59 |  |
| **HDL-C (mmol/L)** | 1.1±0.3 | 1.1±0.2 | 0.99 |  | 1.1±0.2 | 1.2±0.3 | 0.76 |  |
| **LDL-C (mmol/L)** | 3.2±0.7 | 3.1±0.7 | 0.42 |  | 3.1±0.8 | 3.1±0.7 | 0.86 |  |
| **FSG (mmol/L)** | 5.1±0.8 | 5.0±1.0 | 0.34 |  | 5.3±1.7 | 5.3±1.1 | 0.98 |  |
| **HOMA-IR** | 2.4 (1.3, 3.2) | 1.9 (1.4, 2.7) | 0.55 |  | 2.9 (2.0, 4.6) | 2.7 (1.6, 3.8) | 0.26 |  |
| **Uric acid (μmol/L)** | 421±122 | 408±71 | 0.60 |  | 429±103 | 457±124 | 0.13 |  |
| **Liver stiffness (kPa)** | 5.9 (4.9, 6.7) | 6.0 (5.6, 6.5) | 0.45 |  | 6.0 (5.4, 7.2) | 5.8 (5.3, 6.7) | 0.34 |  |
| **Liver fat content (%)** | 18.8 (13.3, 25.6) | 11.5 (6.5, 16.0) | ＜0.001 |  | 15.0 (10.8, 24.0) | 11.2 (8.4, 19.1) | 0.019 |  |
| **Body composition** |  |  |  |  |  |  |  |  |
| **ASM (kg)** | 20.6±4.0 | 20.8±4.1 | 0.79 |  | 25.5±5.6 | 25.5±5.6 | 0.99 |  |
| **ASM/W (%)** | 32.1±4.1 | 33.3±4.1 | 0.17 |  | 31.7±4.0 | 31.±4.2 | 0.99 |  |
| **Body muscle mass (kg)** | 24.4±3.3 | 23.9±3.8 | 0.57 |  | 26.7±3.9 | 26.8±3.8 | 0.88 |  |
| **Total fat mass (kg)** | 15.4±3.7 | 15.4±3.8 | 0.99 |  | 23.3±7.7 | 25.4±11.5 | 0.17 |  |

Continuous variables are reported as mean ± standard deviation (SD) or median (interquartile range).

BMI, body mass index; WC, Waist circumference; ALT, Alanine aminotransferase; AST, Aspartate aminotransferase, GGT, γ-glutamyl transpeptidase; ALP, alkaline phosphatase; TC, total cholesterol; TG, triglyceride; HDL-C, high-density lipoprotein cholesterol; LDL-C, low-density lipoprotein cholesterol; FSG, fasting serum glucose; HOMA-IR, homeostatic model assessment of insulin resistance; ASM, appendicular skeletal mass; ASM/W, ASM/weight.

**Supplementary table 7. Factors associated with improvement of liver fat content* in univariate and multivariate logistic regression analysis in subjects classified by BMI <25kg/m^2^ and BMI≥25kg/m^2^.**

| **Factors** | **BMI <25kg/m^2^** | |  | **BMI≥25kg/m^2^** | |
| --- | --- | --- | --- | --- | --- |
|  | **Univariate** | | **Univariate** | | |
|  | **OR (95% CI)** | ***P*** | **OR (95% CI)** | | ***P*** |
| **Age, years** | 1.02(0.99-1.05) | 0.20 | 1.00(0.98-1.03) | | 0.70 |
| **Male** | 1.41(0.58-3.40) | 0.45 | 1.34(0.65-2.76) | | 0.43 |
| **Hypertension** | 2.04(0.81-5.12) | 0.13 | 0.74(0.39-1.41) | | 0.36 |
| **Diabetes** | 2.26(0.41-12.35) | 0.35 | 1.26(0.57-2.78) | | 0.57 |
| **Smoke** | 1.30(0.34-5.01) | 0.71 | 0.92(0.35-2.43) | | 0.86 |
| **Alcohol consumption** | 0.90(0.21-3.86) | 0.88 | 0.52(0.18-1.46) | | 0.21 |
| **Weight (kg)** | 0.98(0.92-1.05) | 0.55 | 0.98(0.95-1.00) | | 0.09 |
| **BMI, kg/m2** | 0.95(0.69-1.39) | 0.90 | 0.94(0.86-1.03) | | 0.20 |
| **WC, cm** | 0.93(0.85-1.01) | 0.09 | 0.95(0.92-0.99) | | 0.022 |
| **Insulin resistance** | 3.16(1.23-8.09) | 0.017 | 1.00(0.53-1.91) | | 0.99 |
| **ASM/W (%)** | 0.97(0.87-1.07) | 0.51 | 0.96(0.89-1.04) | | 0.33 |
| **Body muscle mass (kg)** | 1.01(0.89-1.14) | 0.93 | 0.93(0.86-1.01) | | 0.09 |
| **Total fat mass (kg)** | 0.95(0.84-1.06) | 0.33 | 0.97(0.94-1.01) | | 0.09 |
| **Decrease in Weight ^a^ (kg)** | 6.21(2.44-15.81) | <0.001 | 7.98(3.86-16.52) | | <0.001 |
| **Decrease in HOMA-IR ^b^** | 4.86(1.95-12.08) | 0.001 | 2.95(1.52-5.74) | | 0.001 |
| **△ASM/W _Baseline-Follow-up_ (%)** | 2.61(1.67-4.07) | <0.001 | 2.33(1.72-3.15) | | <0.001 |
| △**Body muscle mass _Baseline -Follow-up_ (kg)** | 0.44(0.18-1.07) | 0.07 | 0.98(0.76-1.27) | | 0.86 |
| △**Body fat mass _Baseline -Follow-up_ (kg)** | 1.03(0.81-1.30) | 0.81 | 1.84(1.31-2.58) | | <0.001 |

BMI, body mass index; OR, odds ratio; CI, confidence interval; WC, Waist circumference; HOMA-IR, homeostatic model assessment of insulin resistance; ASM, appendicular skeletal mass; ASM/W, ASM/weight.

^a^ Decrease in Weight was defined as △Weight _Baseline-Follow-up_ >0; ^b^ Decrease in HOMA-IR was defined as △HOMA-IR _Baseline-Follow-up_ >0. *The improvement of liver steatosis was defined as the liver fat content (LFC) ≥30% relative decline compared with baseline in MRI-PDFF (LFC _Baseline-Follow-up_/ LFC _Baseline_≥30%).

**Supplementary table 8. Factors associated with improvement of liver fat content* in univariate and multivariate logistic regression analysis in subjects classified by BMI <30kg/m^2^ and BMI≥30kg/m^2^.**

| **Factors** | **BMI <30kg/m^2^ (n=219)** | | | |  | **BMI≥30kg/m^2^ (n=37)** | | | |
| --- | --- | --- | --- | --- | --- | --- | --- | --- | --- |
|  | **Univariate** | | **Multivariate** | | **Univariate** | | | **Multivariate** | |
|  | **OR (95% CI)** | ***P*** | **OR (95% CI)** | ***P*** | **OR (95% CI)** | | ***P*** | **OR (95% CI)** | ***P*** |
| **Age, years** | 1.01(0.99-1.03) | 0.19 |  |  | 0.98(0.93-1.03) | | 0.40 |  |  |
| **Male** | 0.63(0.34-1.16) | 0.14 |  |  | 2.55(0.58-11.28) | | 0.22 |  |  |
| **Hypertension** | 1.08(0.61-1.92) | 0.79 |  |  | 0.91(0.23-3.52) | | 0.89 |  |  |
| **Diabetes** | 1.44(0.67-3.10) | 0.35 |  |  | 1.65(0.26-10.36) | | 0.60 |  |  |
| **Smoke** | 1.04(0.46-2.38) | 0.92 |  |  | 0.83(0.05-14.48) | | 0.90 |  |  |
| **Alcohol consumption** | 0.61(0.25-1.47) | 0.27 |  |  | 1.67(0.14-20.23) | | 0.69 |  |  |
| **Weight (kg)** | 0.99(0.97-1.02) | 0.60 |  |  | 0.96(0.91-1.01) | | 0.14 |  |  |
| **BMI, kg/m2** | 1.06(0.94-1.19) | 0.35 |  |  | 0.75(0.58-0.97) | | 0.029 | 1.56(0.51-4.77) | 0.41 |
| **WC, cm** | 0.97(0.94-1.01) | 0.14 |  |  | 0.95(0.87-1.03) | | 0.18 |  |  |
| **Insulin resistance** | 2.04(1.14-3.65) | 0.017 | 1.12(0.36-3.46) | 0.84 | 0.17(0.02-1.56) | | 0.12 |  |  |
| **ASM/W (%)** | 0.93(0.87-1.00) | 0.06 |  |  | 1.14(0.96-1.35) | | 0.14 |  |  |
| **Body muscle mass (kg)** | 0.95(0.88-1.02) | 0.18 |  |  | 1.04(0.91-1.20) | | 0.58 |  |  |
| **Total fat mass (kg)** | 1.01(0.96-1.06) | 0.75 |  |  | 0.88(0.79-0.98) | | 0.022 | 0.75(0.46-1.22) | 0.24 |
| **Decrease in Weight ^a^ (kg)** | 7.89(4.25-14.65) | <0.001 | 3.13(1.00-9.74) | 0.049 | 4.25(0.98-18.40) | | 0.053 |  |  |
| **Decrease in HOMA-IR ^b^** | 3.36(1.89-5.99) | <0.001 | 6.89(2.14-22.16) | 0.001 | 6.86(1.40-33.57) | | 0.018 | 2.17(0.08-60.10) | 0.65 |
| **△ASM/W _Baseline-Follow-up_ (%)** | 2.73(2.02-3.68) | <0.001 | 3.06(2.14-5.64) | 0.001 | 1.73(1.52-2.61) | | 0.008 | 1.4(0.63-3.30) | 0.39 |
| △**Body muscle mass _Baseline -Follow-up_ (kg)** | 2.00(1.58-2.53) | <0.001 | 1.84(1.04-3.27) | 0.037 | 2.00(1.15-3.49) | | 0.015 | 1.17(0.45-3.08) | 0.75 |
| △**Body fat mass _Baseline -Follow-up_ (kg)** | 1.07(0.98-1.15) | 0.12 |  |  | 1.82(0.74-4.46) | | 0.19 |  |  |

BMI, body mass index; OR, odds ratio; CI, confidence interval; WC, Waist circumference; HOMA-IR, homeostatic model assessment of insulin resistance; ASM, appendicular skeletal mass; ASM/W, ASM/weight.

^a^ Decrease in Weight was defined as △Weight _Baseline-Follow-up_ >0; ^b^ Decrease in HOMA-IR was defined as △HOMA-IR _Baseline-Follow-up_ >0. *The improvement of liver steatosis was defined as the liver fat content (LFC) ≥30% relative decline compared with baseline in MRI-PDFF (LFC _Baseline-Follow-up_/ LFC _Baseline_≥30%).
